# Apoptosis inhibitor of macrophage ameliorates fungus-induced peritoneal injury model in mice

**DOI:** 10.1038/s41598-017-06824-6

**Published:** 2017-07-25

**Authors:** Takako Tomita, Satoko Arai, Kento Kitada, Masashi Mizuno, Yasuhiro Suzuki, Fumiko Sakata, Daisuke Nakano, Emiri Hiramoto, Yoshifumi Takei, Shoichi Maruyama, Akira Nishiyama, Seiichi Matsuo, Toru Miyazaki, Yasuhiko Ito

**Affiliations:** 1 Department of Nephrology and Renal Replacement Therapy, Nagoya University Graduate School of Medicine, Nagoya, Japan; 2 Laboratory of Molecular Biomedicine for Pathogenesis, Center for Disease Biology and Integrative Medicine, Faculty of Medicine, University of Tokyo, Tokyo, Japan; 3 Department of Pharmacology, Kagawa University, Kagawa, Japan; 4 Department of Medicinal Biochemistry, Aichi Gakuin University School of Pharmacy, Nagoya, Japan; 5 AMED-CREST, Japan Agency for Medical Research and Development, Tokyo, Japan; 6 Present Address: Division of Clinical Pharmacology, Vanderbilt University Medical Center, Nashville, TN USA

## Abstract

Fungal peritonitis in a patient on peritoneal dialysis (PD) is a refractory injury accompanied by severe inflammation, predisposing patients to a poor prognosis. Defective clearance of necrotic tissue interferes with amelioration of tissue injury and induces abnormal tissue remodeling. In the recent reports, apoptosis inhibitor of macrophage (AIM, also called CD5L) prevents obesity, hepatocellular carcinoma and acute kidney injury. Here, we investigated potential roles of AIM in prevention of progression of fungal peritonitis models. *AIM* ^−/−^ mice subjected to zymosan-induced peritonitis exhibited progressive inflammation and sustained peritoneal necrosis tissue on day 28 after the disease induction, whereas there was an improvement in *AIM* ^+/+^ mice. This appeared to be caused by deposition of AIM at the necrotic peritoneum in *AIM* ^+/+^ mice. *In vitro*, AIM enhanced the engulfment of necrotic debris by macrophages derived from zymosan-induced peritonitis, M1- and M2a-like bone marrow derived macrophages, as well as by mesothelial cells. In addition, administration of recombinant AIM dramatically ameliorated severe inflammation associated with necrosis in zymosan-induced peritonitis of *AIM* ^−/−^ mice. Our observations suggest that AIM appears to be involved in the repair process of zymosan-induced peritonitis, and thus, could be the basis of development of new therapeutic strategies for PD-related fungal peritonitis.

## Introduction

Infective peritonitis is one of the most important complications in patients on peritoneal dialysis (PD) therapy^1, 2^, which induces angiogenesis, lymphangiogenesis and fibrosis, leading to peritoneal membrane dysfunction^3–8^. Although fungal peritonitis is not common, it has the most severe and poor prognosis due to the associated severe inflammation^8–10^. Encapsulating peritoneal sclerosis (EPS), which is a life-threatening complication of PD involving peritoneal membrane deterioration, usually develops after long-term PD treatment^11–13^. However, the severe inflammation associated with even a single episode of fungal peritonitis can reportedly induce EPS^14–17^. Therefore, prevention and minimization of inflammation in this condition is vital.

In recent years, peritonitis is still a serious complication despite the common use of sterile connecting systems for bag exchange in patients on PD^18, 19^. Development of peritonitis is considered to be related to the condition of the host defense systems. Presence of diabetes^20, 21^, malnutrition^22^, low serum albumin levels^20, 23^ and low serum 25-hydroxyvitamin D concentration^24^ have all been reported to affect the defense system against microorganisms in PD patients.

Decrease in the clearance of debris, such as apoptotic or necrotic cells, has been reported to prevent resolution of inflammation and tissue remodeling, leading to fibrosis and organ dysfunction^25–29^. In particular, necrotic cells are reported to induce more severe inflammation than apoptotic cells. Not only macrophages, but also bronchial epithelial cells^27^, mammary epithelial cells^30^ and mesothelial cells^31^, are involved in the engulfment of debris and tumor cells. Bacterial or fungal infection reportedly induced necrosis^32, 33^. Apoptosis inhibitor of macrophage (AIM, also called CD5L), a member of the scavenger receptor cysteine-rich superfamily, is a circulating protein initially identified as a supporter of macrophage survival^34, 35^, which has been shown to be endocytosed by macrophages, adipocytes, hepatocytes and tubular epithelial cells^36–40^. Recently, we reported that AIM enhances clearance of intraluminal debris in acute kidney injury (AKI) mainly by tubular epithelial cells^29^. In addition, AIM has been suggested to exert antimicrobial activity by causing clumping of bacteria or fungus, thus enhancing these clearance^38–40^.

We previously reported a zymosan-induced peritonitis model (zymosan model), which is a complement-dependent fungus-induced peritonitis model with similar clinicopathology as human fungal peritonitis, including Candida peritonitis^1, 41–43^. However, although activation of complement is involved in the onset of the fungal peritonitis model, inflammation cannot be completely suppressed by the inhibition of complement alone, suggesting that other factors seem to be involved in the disease development.

Macrophages are largely classified into two functional subtypes. Classical active macrophages, M1 macrophages (representative marker: iNOS) are defined by their cytotoxic properties, while M2 macrophages (representative marker: CD206) are characterized by anti-inflammatory and regulatory properties^44^. In the zymosan model, it is reported that there is infiltration of both M1 and M2 macrophages^45^, although no studies have described the transition in detail. Further, although macrophages are reportedly involved in the clearance of debris of neutrophils (CD11b^+^Ly6B2^+^)^45^, whether this is mainly a contribution of M1 or M2 macrophages remains unknown. Mannose receptor (CD206) positive macrophages recognize and endocytose microorganisms^46^. However, the types of macrophages that modify the changes in disease progression and regression in the zymosan model still remain obscure.

In this study, we investigated the protective effects of AIM in a zymosan model and the possible preventive roles of AIM in PD-related peritonitis by clearance of necrotic tissue and debris in the peritoneal cavity.

## Results

### Zymosan-induced peritonitis was less severe in wild mice than in AIM-deficient mice

**Figure 1:**
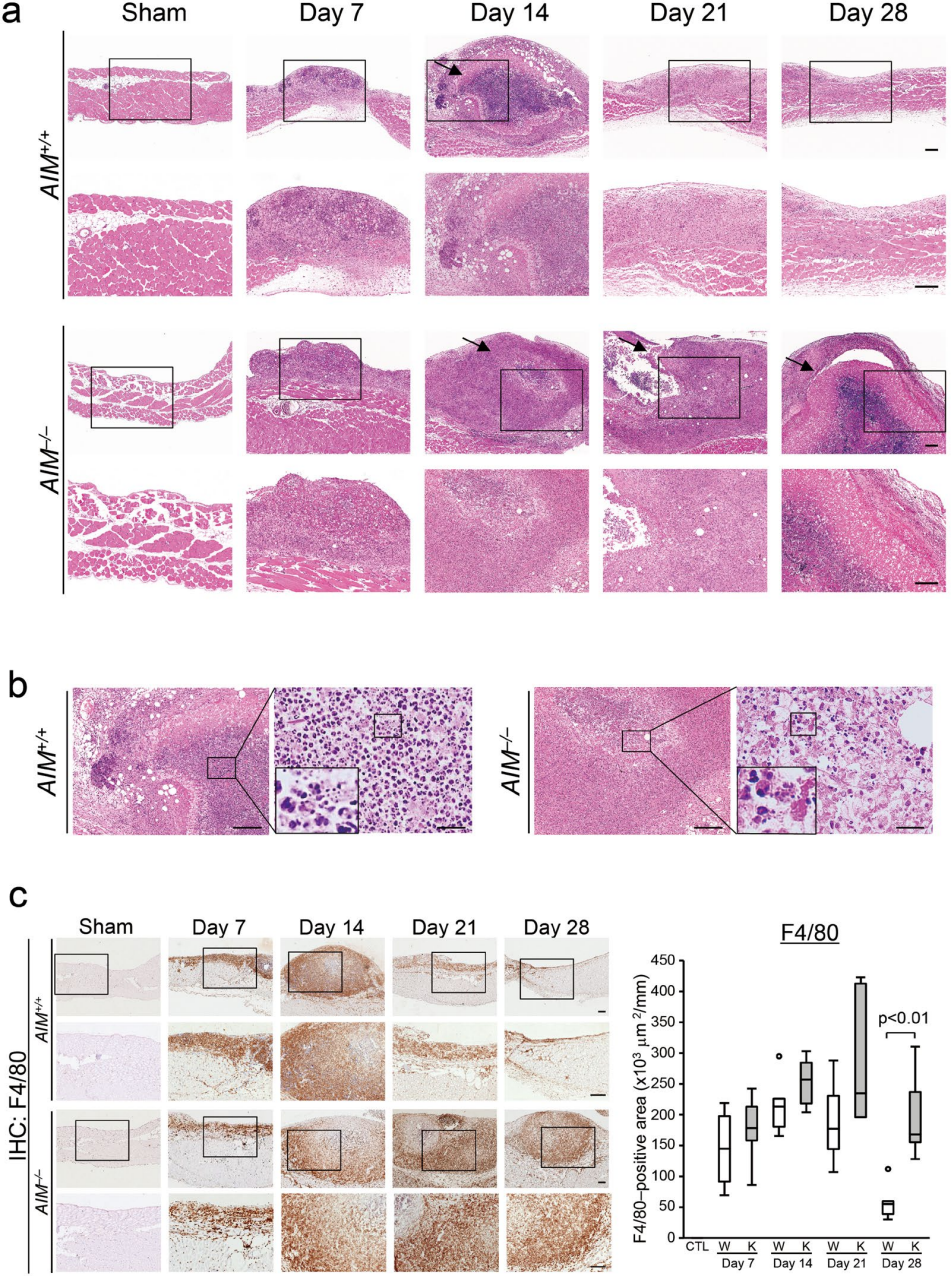
Morphological changes in the zymosan-induced peritonitis model. (**a**) Strong inflammatory cell infiltration associated with a necrotic area was demonstrated up to 4 weeks in AIM-deficient mice; in contrast, these changes subsided at 3 weeks after disease induction in the wild mice. The first and second rows: *AIM* ^+/+^ mice, The third and fourth rows: *AIM* ^−/−^ mice; The second and fourth rows show higher magnifications of the images in the boxed areas of the first and third rows, respectively. HE stain, arrows: necrotic area, Scale bars, 200 μm. Sham and Day 7–21: *n* = 6, Day 28: *n* = 14. (**b**) Light microscopic findings indicated necrosis associated with strong neutrophilic infiltration with karyorrhexis. The figures are enlarged views of day 14 in Fig. 1a. The left two photographs present the findings in *AIM* ^+/+^ mice, and the right two in *AIM* ^−/−^ mice. The insets in the large magnified photos indicate karyorrhexis. Karyorrhexis is a degenerative cellular process characterized by fragmentation of the condensed nucleus and irregular distribution of the resultant chromatin in the cytoplasm^47^. HE stain, Scale bars, 200 μm (each left figure) and 20 μm (each right figure). (**c**) Expression of F4/80 positive macrophages in the zymosan-induced peritonitis model. Macrophage infiltration continued up to 4 weeks in AIM-deficient mice, while it decreased 3 weeks after disease induction in the wild mice. Left figures: The second and fourth rows show higher magnifications of the images in the boxed areas in the corresponding images in the first and third rows, respectively. Right graphs: Macrophage positive areas were assessed by morphometry and were expressed as ×10^3^ μm^2^/mm surface length. W: *AIM* ^+/+^ mice; K: *AIM* ^−/−^ mice; CTL: control normal *AIM* ^+/+^ mice, Scale bars, 200 μm. *n* = 6 for each group.

In order to address the hypothesis that AIM is involved in the susceptibility to peritonitis, particularly fungal peritonitis, we investigated wild type (*AIM* ^+/+^) and AIM-deficient (*AIM* ^−/−^) zymosan model mice (Supplementary Figure 1a). In the *AIM* ^−/−^ mice, strong inflammation with necrosis continued for up to day 28 after disease induction, whereas the peritoneum in the *AIM* ^+/+^ mice showed signs of resolution in the form of tissue regeneration (Fig. 1a). The presence of a necrotic area on day 28 was seen more often in *AIM* ^−/−^ mice than in *AIM* ^+/+^ mice (1 of 14 mice in the *AIM* ^+/+^ mice group and 11 of 13 mice in the *AIM* ^−/−^group, p < 0.001). The necrotic area was often associated with karyorrhexis of neutrophils (Fig. 1b)^47^. These findings indicate that a deficiency of AIM apparently worsens the pathology, resulting in severe necrosis and inflammation in the zymosan model.

### Expression of surface markers of macrophages and pro-inflammatory cytokines in zymosan models assessed by immunohistochemistry and quantitative PCR

**Figure 2:**
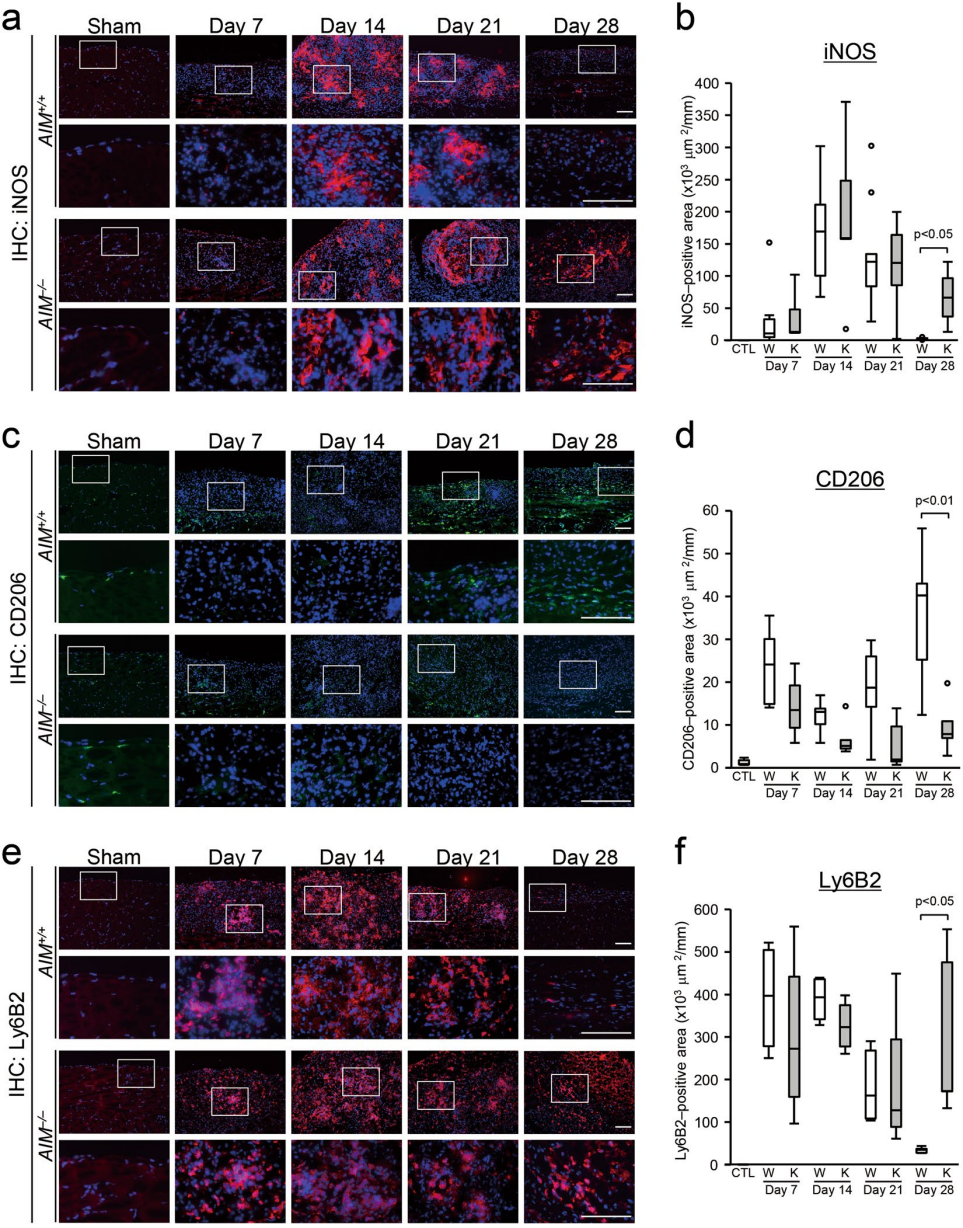
Expression of surface markers of inflammatory cells in the zymosan-induced peritonitis model. Immunostaining of the peritoneal wall for iNOS (**a**,**b**), CD206 (**c**,**d**) and Ly6B2 (**e**,**f**). On day 28, the number of iNOS positive macrophages with M1 polarity ((**a)** red color) and Ly6B2 positive cells ((**e)** red color) was significantly higher in *AIM* ^−/−^ mice than *AIM* ^+/+^ mice, while CD206-positive macrophage expression with M2 polarity ((**c**) green color) was higher in *AIM* ^+/+^ mice compared with *AIM* ^−/−^ mice. The nuclei were counterstained with 4′, 6-diamidino-2-phenylindole (DAPI, blue color). The second and fourth rows show higher magnifications of the images in the boxed areas of the first and third rows, respectively. Positive areas were assessed by morphometry (**b**,**d** and **f**). W: *AIM* ^+/+^ mice; K: *AIM* ^−/−^ mice; CTL: control normal *AIM* ^+/+^ mice, Scale bars, 100 μm. *n* = 6 for each group.

**Figure 3:**
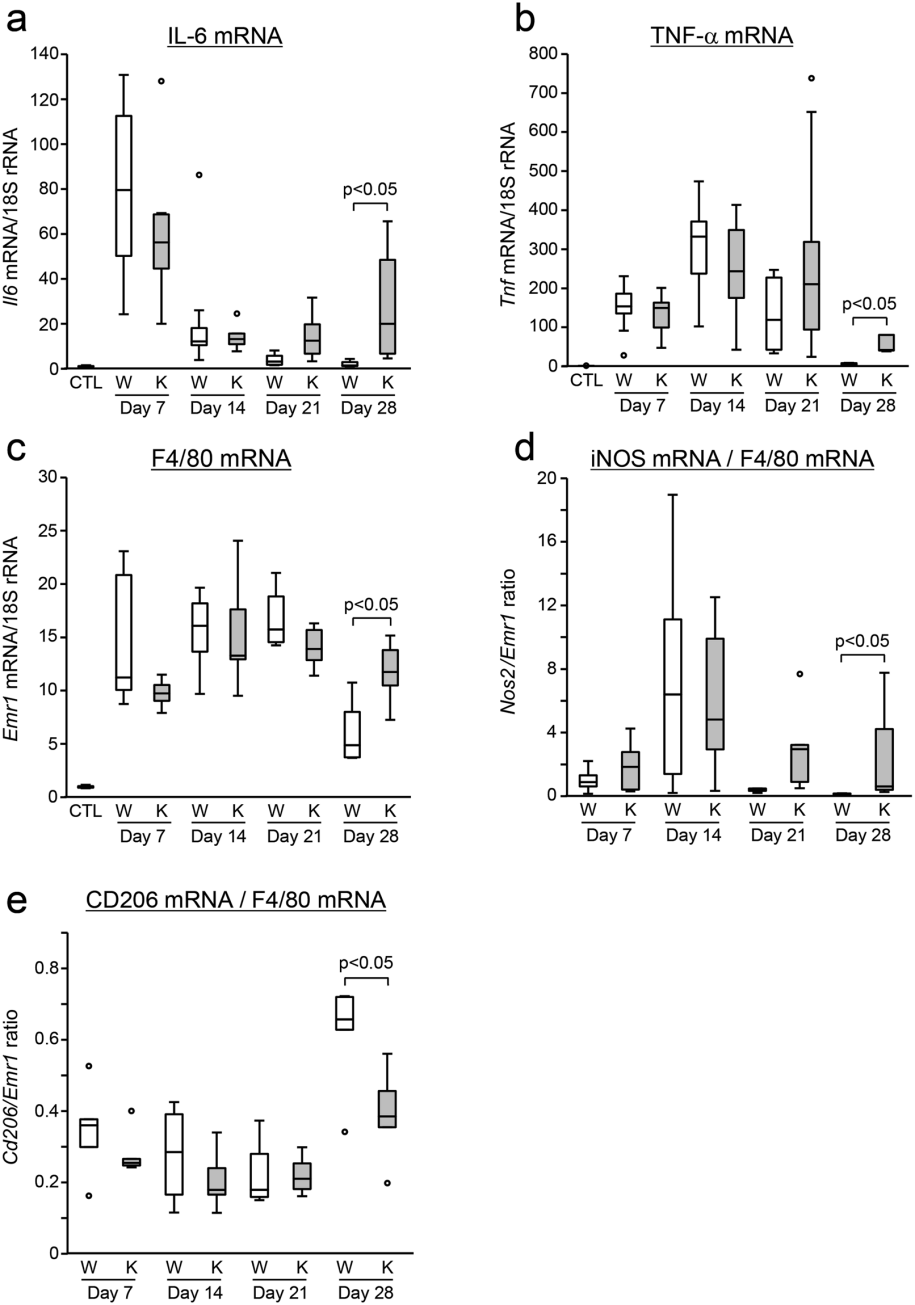
Expression of inflammatory markers in the zymosan-induced peritonitis model, as assessed by quantitative real-time PCR. (**a**) *Il6* (IL-6) mRNA, (**b**) *Tnf* (TNF-α) mRNA, (**c**) *Emr1* (F4/80) mRNA, (**d**) *Nos2* (iNOS) mRNA/*Emr1* (F4/80) mRNA, (**e**) *Cd206* (CD206) mRNA/*Emr1* (F4/80) mRNA. W: *AIM* ^+/+^ mice; K: *AIM* ^−/−^ mice, CTL: control normal *AIM* ^+/+^ mice. *n* = 6 for each group.

In order to compare the inflammatory conditions between *AIM* ^+/+^ and *AIM* ^−/−^ mice, we investigated the surface markers of inflammatory cells and pro-inflammatory cytokines. CD11b and F4/80 positive macrophage infiltration was increased from day 7 and continued past day 28 in the zymosan model *AIM* ^−/−^ mice. On day 28, infiltration of CD11b and F4/80 positive macrophages was significantly higher in the *AIM* ^−/−^ mice than *AIM* ^+/+^ mice (p < 0.01, Fig. 1c and Supplementary Figure 2 and Supplementary Table 1). Inducible nitric oxide synthase (iNOS) positive macrophages (M1-like macrophages) were predominant on day 14, tending to decrease thereafter in both *AIM* ^+/+^ and *AIM* ^−/−^ mice. However, on day 28, iNOS positive macrophage infiltration was significantly higher in the *AIM* ^−/−^ mice than *AIM* ^+/+^ mice (p < 0.05, Fig. 2a,b and Supplementary Table 1). In contrast, CD206 positive macrophages (M2-like macrophages) were predominant on days 21 and 28 in *AIM* ^+/+^ mice. CD206 positive macrophage infiltration was significantly higher in *AIM* ^+/+^ mice than in *AIM* ^−/−^ mice on day 28 (p < 0.05, Fig. 2c,d and Supplementary Table 1). There was strong infiltration of lymphocyte antigen 6 complex and locus B2 (Ly6B2) positive cells on days 7 and 14, tending to subside thereafter in the *AIM* ^+/+^ mice. Ly6B2 positive cells infiltration was also significantly higher in the *AIM* ^−/−^ mice than *AIM* ^+/+^ mice on day 28 (p < 0.05, Fig. 2e,f and Supplementary Table 1). There was slight infiltration of CD4, CD8 and CD11c positive cells, but there were no significant differences between the groups (Supplementary Figure 3). Interleukin (IL)-6, tumor necrosis factor (TNF)-α and F4/80 mRNA expression was significantly higher on day 28 in *AIM* ^−/−^ mice compared with *AIM* ^+/+^ mice (p < 0.05, Fig. 3a–c). The relative ratio of iNOS mRNA/F4/80 mRNA was also higher in *AIM* ^−/−^ mice. In contrast, the ratios of CD206 mRNA/F4/80 mRNA and CD163 mRNA/F4/80 mRNA were significantly higher in the *AIM* ^+/+^ mice on day 28 (p < 0.05, Fig. 3d,e and Supplementary Figure 4). These findings indicate that on day 28, inflammatory cytokines and expression of M1-like macrophages were higher in *AIM* ^−/−^ mice, while M2-like macrophages were predominant in *AIM* ^+/+^ mice.

### Expression of AIM increased in zymosan-induced peritonitis

**Figure 4:**
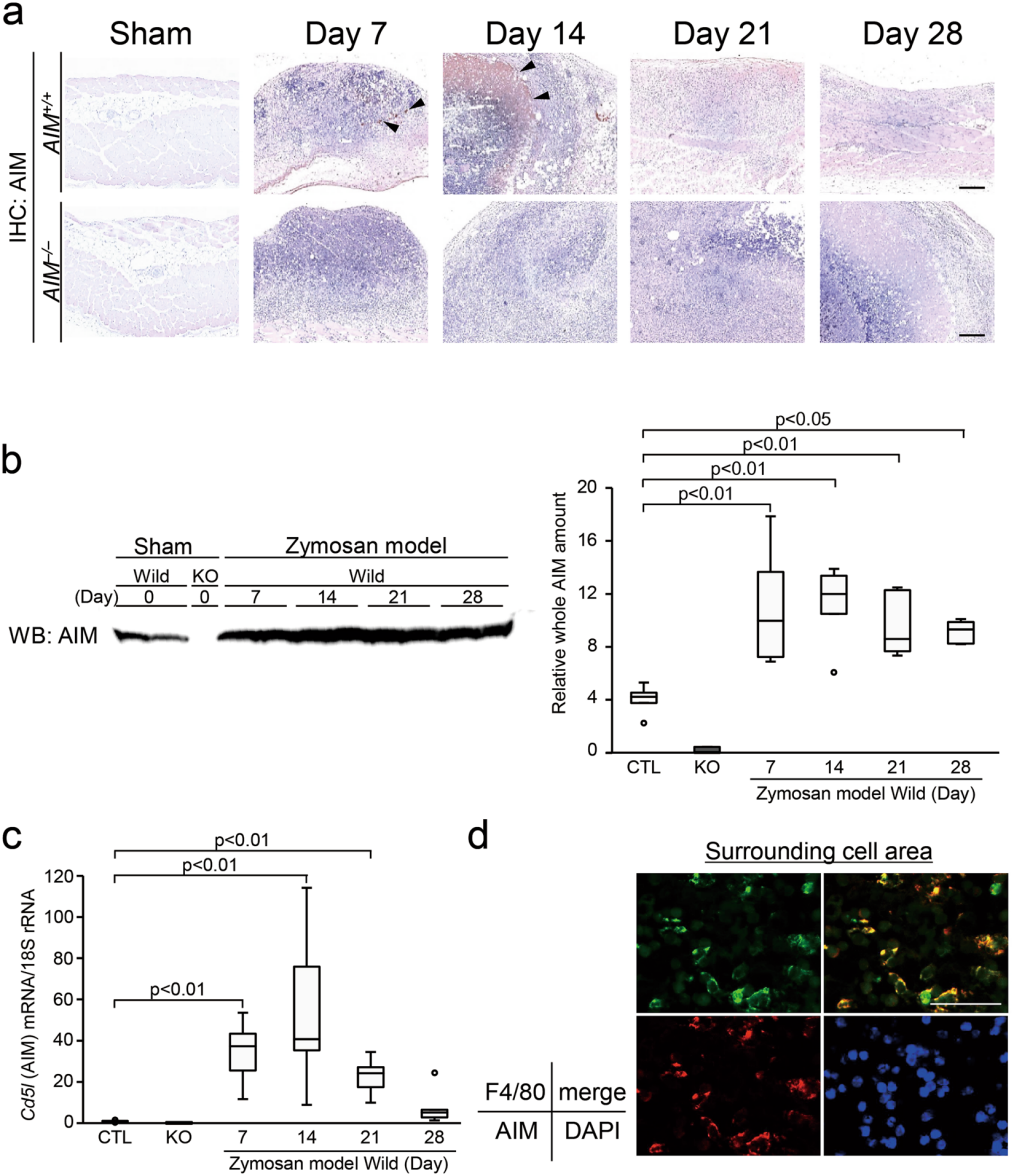
Expression of AIM in the zymosan-induced peritonitis model. Expression of AIM was increased in the wild mice. (**a**) Immunohistochemistry revealed AIM in the area of peritonitis in *AIM* ^+/+^ mice, this being most abundant in the necrotic areas (arrow heads). There was no expression of AIM in *AIM* ^−/−^ mice. Scale bars, 200 μm. (**b**) Western blotting revealed an increase in circulating AIM in serum from day 7 in zymosan model *AIM* ^+/+^ mice. (**c**) *Cd5l* (AIM) mRNA assessed by real-time PCR was upregulated from day 7 in zymosan model *AIM* ^+/+^ mice. CTL: control normal *AIM* ^+/+^ mice, KO: *AIM* ^−/−^ mice. *n* = 6 for each group. (**d**) Double staining of F4/80 (green) and AIM (red) revealed that AIM was released from macrophages in the surrounding cell area. Blue indicates DAPI staining. Scale bar, 50 μm.

Immunohistochemistry demonstrated AIM in the area of peritonitis, this being most obvious in the necrotic area (Fig. 4a). Western blotting revealed an increase in circulating AIM levels in serum (Fig. 4b). AIM mRNA expression in the peritoneal wall also increased in *AIM* ^+/+^ mice, and mainly expressed by macrophages (Fig. 4c,d).

### Complement activation in the acute phase of the disease was not different between the groups

Since initiation of zymosan-induced peritonitis is complement dependent, due to direct activation of alternative pathways by zymosan^1^, we assessed C3 and membrane attack complex (C5b-9) deposition in the peritoneal wall. There was no difference in the activation of complement in the early stages of peritonitis between *AIM* ^+/+^ and *AIM* ^−/−^ mice (Supplementary Figure 5).

### Administration of recombinant AIM ameliorated peritoneal inflammation in zymosan model AIM^−/−^ mice

**Figure 5:**
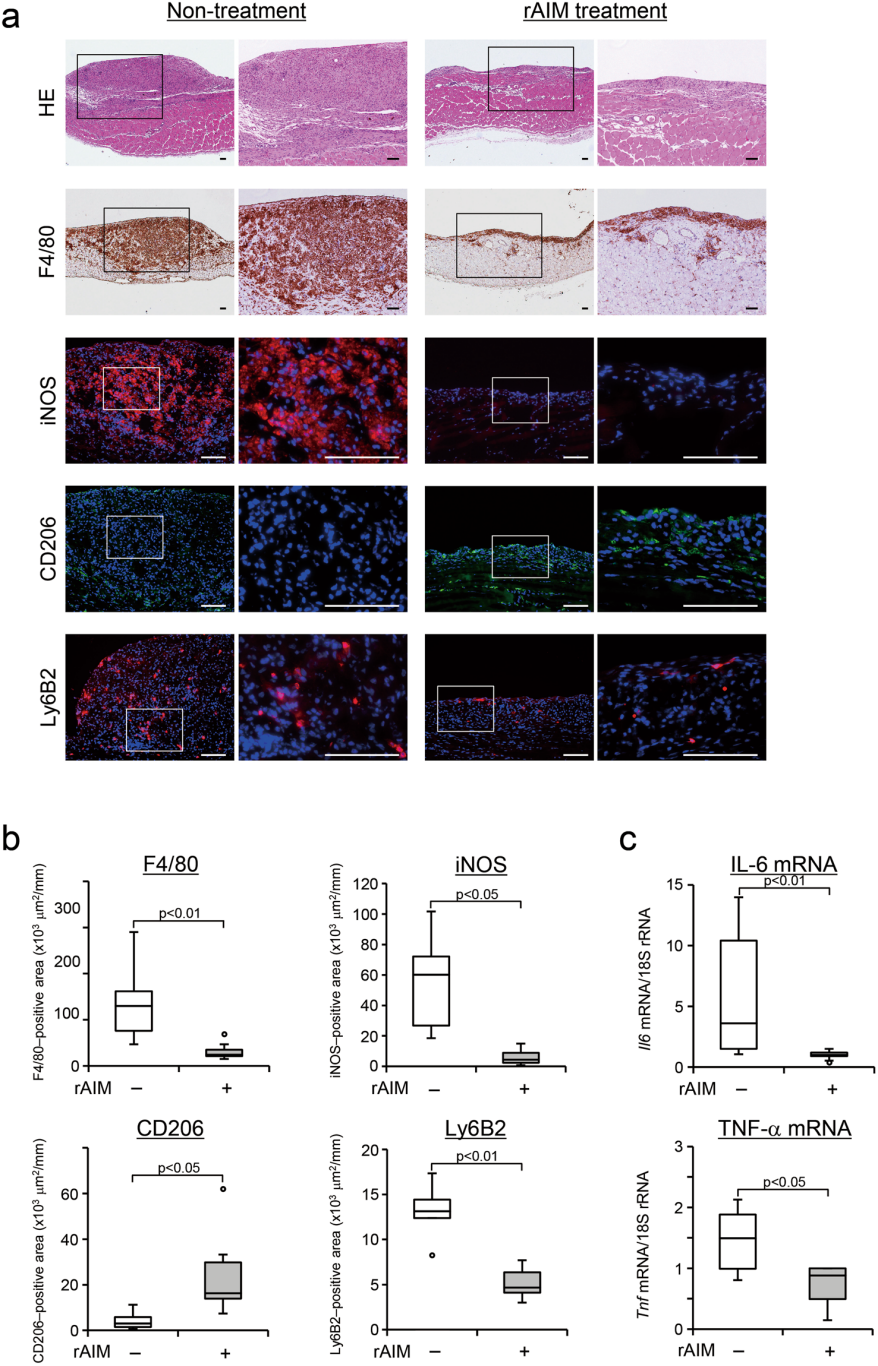
Administration of recombinant AIM reduced macrophage infiltration in *AIM* ^−/−^ mice of the zymosan-induced peritonitis model. (**a**,**b**) We assessed the expression of F4/80, iNOS, CD206 and Ly6B2 using immunohistochemistry. Recombinant AIM (rAIM) suppressed F4/80-positive macrophage infiltration at 4 weeks in zymosan model *AIM* ^−/−^ mice when compared with control group. In addition, iNOS and Ly6B2 positive cells were significantly suppressed in the AIM treatment group. In contrast, CD206 expression was higher in the AIM treatment group. Right picture shows higher magnifications of the images in the boxed areas of the left picture, respectively. The nuclei were counterstained with DAPI (blue color). (**c**) We assessed the expression of IL-6 and TNF-α using real-time PCR. IL-6 and TNF-α mRNA expression was reduced by administration of AIM. rAIM: rAIM treatment, Scale bars, 100 μm. *n* = 11 for each group.

We next addressed whether recombinant AIM (rAIM) administration can suppress inflammation in the zymosan model *AIM* ^−/−^ mice. rAIM suppressed inflammation with necrosis on day 28 in the zymosan model *AIM* ^−/−^ mice compared with the non-treatment group. These effects were associated with suppression of F4/80 (p < 0.01) positive macrophages, iNOS positive macrophages (p < 0.05), Ly6B2 positive cells (p < 0.01), IL-6 (p < 0.01) and TNF-α (p < 0.05) mRNA expression (Fig. 5). In contrast, CD206 positive macrophage levels were significantly higher in the rAIM treatment group (p < 0.05, Fig. 5).

### Coating with AIM promoted engulfment of necrotic debris by F4/80 positive macrophages derived from the zymosan model, and M1- and M2a-like macrophages

**Figure 6:**
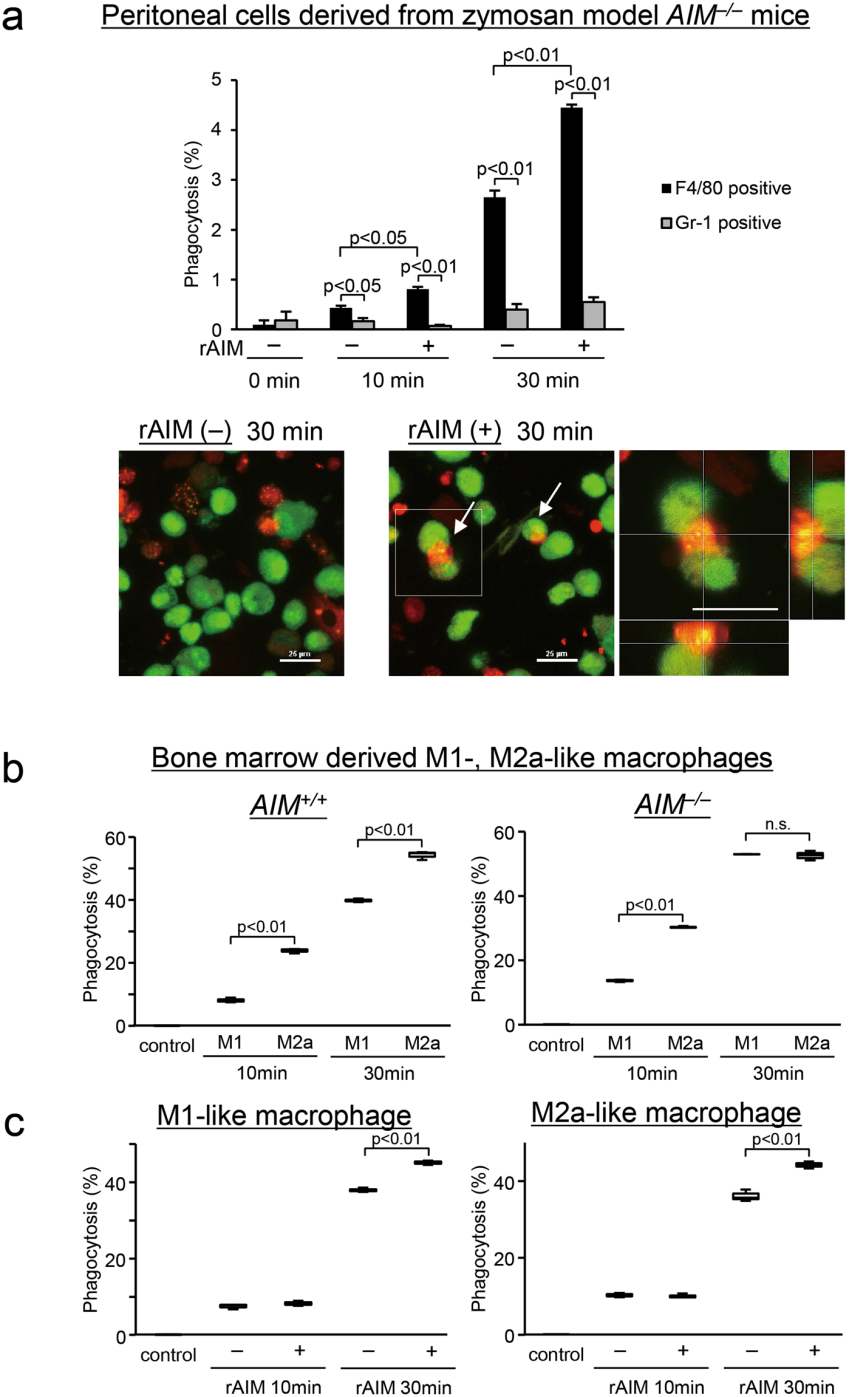
Coating with AIM enhanced debris engulfment by F4/80 positive, M1- and M2a-like macrophages. (**a**) Peritoneal cells derived from zymosan model *AIM* ^−/−^ mice. Phagocytic assay using cells collected from the peritoneum of zymosan model mice on day 7. The proportion of engulfment of FVD780-positive cell debris within DAPI^−^Mac-1^+^F4/80^+^Gr-1^−^ cells and DAPI^−^Mac-1^+^F4/80^−^Gr-1^+^ cells was assessed by flow cytometry. Precise methods are described in the Methods section. F4/80 positive macrophages had a greater phagocytic ability than Gr-1 positive neutrophils at 10 and 30 min. F4/80 positive macrophages had a significantly higher engulfing ability for rAIM coated dead cell debris than for non-coated dead cell debris. *n* = 3 dishes for each group. Confocal microscopic images of engulfment by F4/80 positive macrophages (red) of labeled dead cell debris (green) with and without a coating of rAIM. Phagocytosis by F4/80 positive macrophages was enhanced by coating the surface of the debris with rAIM. Yellow colors (merge) indicates that the dead cell debris engulfed by the macrophages (red). The arrows indicate phagocytosis. Supplementary Videos 1 and 2 provide more precise images of phagocytosis by M1- or M2a-like macrophages. Scale bars, 25 μm. (**b**,**c**) M1- and M2a-like macrophages modified from bone marrow cells. (**b**) M2a-like macrophages from *AIM* ^+/+^ mice phagocytosed dead cell debris to a greater extent than M1-like macrophages at 10 and 30 min after incubation. At 10 min, M2a-like macrophages from *AIM* ^−/−^ mice phagocytosed dead cell debris to a greater extent than M1-like macrophages. *n* = 3 dishes for each group. (**c**) Phagocytosis by both M2a- and M1-like macrophages from *AIM* ^−/−^ mice at 30 min was enhanced by coating the surface of the dead-cell debris with rAIM. Control: Dead cell debris was not incubated, rAIM: Dead cell debris was coated with rAIM. *n* = 3 dishes for each group.

Next, we investigated which type of cells phagocytosed necrotic debris, and the effects of AIM in promoting phagocytosis. We isolated peritoneal cells from zymosan model *AIM* ^−/−^ mice, and examined the phagocytic activity to Fixable Viability Dye eFluor 780 (FVD780, eBioscience, San Diego, CA)-labeled dead cell debris with or without rAIM coating using flow cytometry. F4/80 positive macrophages engulfed labeled debris to a greater extent than Gr-1 positive neutrophils at 10 and 30 min (Fig. 6a). In addition, coating with rAIM enhanced engulfment by macrophages compared with the control group (10 min: p < 0.05, 30 min: p < 0.01) (Fig. 6a).

In order to identify the type of macrophages that are mainly responsible for ingestion of dead cell debris, we differentiated bone marrow cells from both *AIM* ^−/−^ and *AIM* ^+/+^ mice into M1- and M2a-like macrophages and examined their ability to engulf dead cell debris (Supplementary Videos 1 and 2). Flow cytometry revealed that M2a-like macrophages from *AIM* ^−/−^ mice had a significantly greater engulfing ability than M1-like macrophages derived from *AIM* ^−/−^ mice at 10 min (p < 0.01, Fig. 6b and Supplementary Figure 6a). Similar results were observed when *AIM* ^+/+^ mice were used (p < 0.01, Fig. 6b). These findings indicate that M2a-like macrophages have a greater ability to phagocytose necrotic debris than M1-like macrophages. Finally, we assessed whether phagocytosis by M1- or M2a-like macrophages is enhanced by coating the necrotic debris with rAIM. Interestingly, in both M1- (p < 0.01) and M2a-like (p < 0.01) macrophages from *AIM* ^−/−^ mice, addition of rAIM enhanced engulfment at 30 min (Fig. 6c and Supplementary Figure 6b,c). Similar results were observed for both M1- and M2a-like macrophages from *AIM* ^+/+^ mice as well (Supplementary Figure 7). These findings indicate that supplementation of AIM promotes engulfment of necrotic debris by macrophages independent of phenotype.

### Coating with AIM promoted engulfment of dead cell debris by mesothelial cells

**Figure 7:**
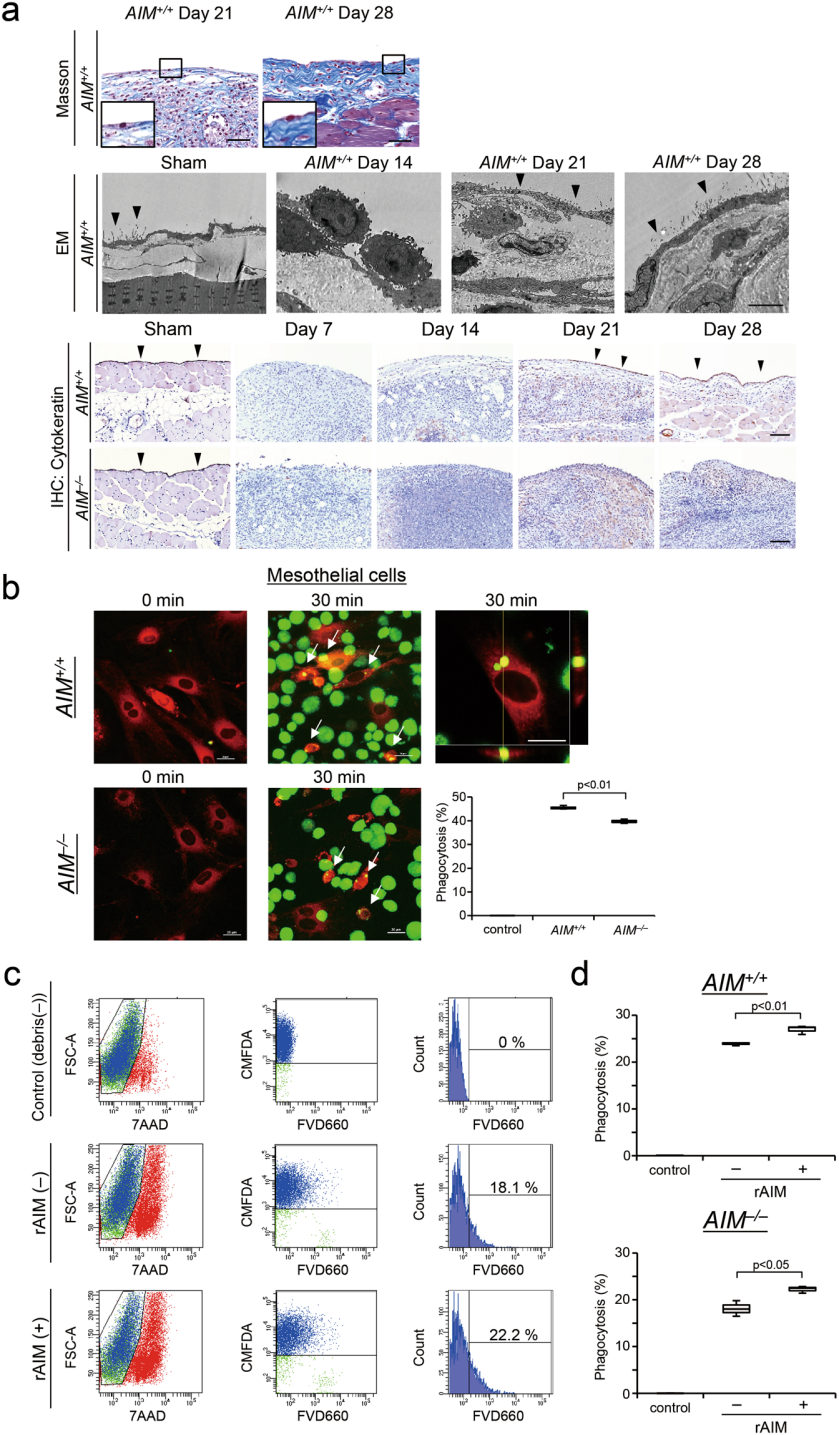
Coating with AIM enhanced debris engulfment by mesothelial cells. (**a**) Masson’s trichrome stain. Mesothelial cells re-appeared on the surface of the peritoneum on days 21 and 28 in *AIM* ^+/+^ mice, respectively. The insets are large magnifications of the boxed areas in the respective figures. Scale bars, 100 μm. Findings of electron microscopy of *AIM* ^+/+^ mice. Sham operation mice: Mesothelial cells were observed on the surface of the peritoneum. Zymosan model *AIM* ^+/+^ mice on day 14: Macrophages were observed on the surface of the peritoneum. Zymosan model *AIM* ^+/+^ mice on days 21 and 28: Mesothelial cells were revealed on the surface of the peritoneal membrane (arrow heads). Scale bar, 5 μm. Immunostaining for cytokeratin. Cytokeratin positive mesothelial cells were observed on the surface of the peritoneum of sham operation mice. Cytokeratin positive mesothelial cells re-appeared on the surface of the peritoneal membrane of the *AIM* ^+/+^ mice (arrow heads) on days 21 and 28. In contrast, mesothelial cells were not observed in the *AIM* ^−/−^ mice. Scale bars, 100 μm. (**b**) Confocal microscopic images showed phagocytosis by mesothelial cells from both *AIM* ^+/+^ and *AIM* ^−/−^ mice. Mesothelial cells from *AIM* ^+/+^ mice had a higher ability of phagocytosis. The arrows indicate phagocytosis. Red: mesothelial cells, Green: dead cell debris, *n* = 3 dishes for each group, Scale bars, 25 μm. Supplementary Video 3 provides more precise images of phagocytosis by mesothelial cells. (**c**,**d**) Cultured mesothelial cells were stained by CellTracker™ Green CMFDA Dye, and mixed with debris labeled by FVD660 with or without rAIM coating. The proportion of engulfment of FVD660 positive dead cell debris by 7-Amino-actinomycin D (7AAD) negative macrophages was assessed. Phagocytosis of mesothelial cells from both *AIM* ^+/+^ and *AIM* ^−/−^ mice was enhanced by coating the debris with rAIM at 90 min. (C) indicates the flow cytometry using mesothelial cells from *AIM* ^−/−^ mice. *n* = 3 dishes for each group. Control: Dead cell debris was not incubated, rAIM: Dead cell debris was coated with rAIM.

Using electron microscopy and immunohistochemistry for cytokeratin, we observed that mesothelial cells reappeared on the surface of the peritoneal membrane during the recovery phase in *AIM* ^+/+^ mice (Fig. 7a). In particular, electron microscopy revealed the presence of macrophages on the surface of the peritoneum on day 14, while mesothelial cells were revealed on days 21 and 28 (Fig. 7a). Thus, in order to investigate whether mesothelial cells contribute to regeneration of the peritoneal membrane in *AIM* ^+/+^ mice, we performed similar phagocytic assays using mouse cultured mesothelial cells, from which CD11b macrophages were depleted by CD11b beads. The purity of mesothelial cells was more than 98%, as verified by flow cytometric analysis and immuno-histochemistry for cytokeratin, CD11b and α-smooth muscle antigen. We found that mouse mesothelial cells engulfed debris (Supplementary Video 3). In addition, mesothelial cells from *AIM* ^+/+^ mice had a higher ability of phagocytosis than those from *AIM* ^−/−^ mice (p < 0.01, Fig. 7b). Importantly, rAIM enhanced phagocytosis by mesothelial cells from both *AIM* ^−/−^ (p < 0.05) and *AIM* ^+/+^ (p < 0.01) mice compared with no treatment (Fig. 7c,d). These findings suggest that mesothelial cells are involved in recovery from inflammation in the late phase in the zymosan-induced peritonitis model.

### Low serum AIM levels in patients who have previously experienced PD-related peritonitis

Finally, we examined the association between AIM and PD peritonitis using human specimens. Despite the careful bag-exchanges using ultraviolet (UV) exposure-sterilized connecting systems during PD, some patients developed peritonitis for unknown reasons. To assess whether low serum AIM levels were related to the susceptibility to peritonitis, we analyzed serum AIM levels in patients with chronic kidney disease on peritoneal dialysis. There was no significant difference in demographic data, including gender, age, presence of diabetes as the primary cause of renal failure, usage of the automated PD cycler machine, serum albumin concentration, protein concentrations in PD effluent at peritoneal equilibration tests (PET), peritoneal transport rate and residual renal function between the peritonitis occurrence and non-occurrence groups (Supplementary Table 2). All patients used connecting equipment sterilized by UV exposure, and the occurrence rate of peritonitis in this cohort was about 0.1 patient^−1^ × year^−1^, which indicates a low incidence of peritonitis at our institute. As shown in Supplementary Figure 8a and b, serum AIM levels were significantly lower in patients who had previously experienced peritonitis than in patients without peritonitis (p < 0.01). Sub-analysis according to gender and age also indicated a higher risk of peritonitis in patients with low serum AIM levels (Supplementary Figure 8c–e), suggesting that AIM may have some protective role to play against peritonitis in PD patients.

## Discussion

Acute inflammation is characterized by infiltration of neutrophils and macrophages. Removal of harmful agents derived from dead cells is necessary to resolve tissue injury and to avoid chronic inflammation^25, 27–30, 48, 49^. In the early stages of peritonitis, the zymosan models showed severe infiltration of macrophages and neutrophils, associated with cell debris and karyorrhexis (Figs 1 and 2 and Supplementary Figure 2)^47^, which resulted in release of proinflammatory cytokines (Fig. 3a,b). These pathological findings are closely related to the concept and pathophysiological hypothesis of necroinflammation and complement activation being an initial trigger in the development of injury^1, 50^. Absence of a difference in complement activation (Supplementary Figure 5) and no significant differences in inflammatory cytokines at 1 week between the groups (Fig. 3a,b) suggest that exacerbation of inflammation in *AIM* ^−/−^ mice was probably due to impairment of clearance rather than induction of necrosis. These findings suggest that absence or insufficient amounts of AIM may cause persistent inflammation in the peritoneum, owing to the impaired clearance of necrotic debris. These findings are consistent with the greater occurrence of peritonitis in human PD patients with lower serum concentrations of AIM (Supplementary Figure 8).

We identified macrophages as being the main professional phagocytes^31^ by phagocytic assay using F4/80 positive macrophages from the zymosan model *AIM* ^−/−^ mice on day 7 (Fig. 6a). Interestingly, M2-like macrophages, which were expressed to a greater extent in *AIM* ^+/+^ mice on day 28 (Fig. 2c,d), tended to engulf the dead cell debris to a greater extent than M1-like macrophages in phagocytic assay (Fig. 6b). These findings suggest that M2-like macrophages are the main players in the reduction of inflammation, as shown by the fact that administration of rAIM promoted recovery from severe inflammation in *AIM* ^−/−^ mice, in association with higher expression of CD206 positive macrophages (Fig. 5a,b). We used IL-4 and IL-13 treated and lipopolysaccharide (LPS) and interferon (IFN)-γ treated macrophages as M2a with higher expression of CD206 or M1-like macrophages with higher expression of CD86, respectively. The M1/M2 paradigm provides us with a useful framework; however, overlapping effects have been indicated^44, 51^. Importantly, AIM effectively enhanced ingestion of dead cell debris not only by M2a-like macrophages, but also by M1-like macrophages, independent of the expression of CD206. Higher engulfment of AIM coated debris is probably due to endocytosis by macrophages via scavenger receptor CD36, which has been previously reported as one of the receptors of AIM^34^.

In the late stages of zymosan-induced peritonitis, mesothelial cells were redistributed on the surface of the peritoneal membrane in *AIM* ^+/+^ mice (Fig. 7a). Mesothelial cells are not professional phagocytes. However, in our experiments, mesothelial cells apparently phagocytosed cell debris (Supplementary Video 3 and Fig. 7b), as also previously reported^31^. Furthermore, AIM enhanced engulfment of dead cell debris by mesothelial cells (Fig. 7c,d). These findings suggest that mesothelial cells seem to be involved as semi-professional phagocytes in promotion of the clearance of debris in the peritoneum^31^, but only in the late stages of zymosan-induced peritonitis models. Mesothelial cells reportedly phagocytose higher levels of dead tumor cells than viable tumor cells^31^, and apparently have phagocytic receptors such as Brain-specific angiogenesis inhibitor 1, T-cell immunoglobulin and mucin domain 1 (Kidney injury molecule 1, KIM-1), Stabilin-2, and scavenger receptors, including lectin-like oxidized LDL receptor-1, CD68 and CD36. Interestingly, addition of serum enhanced ingestion of dead cells by mesothelial cells^31^, suggesting that circulating AIM could be a soluble bridging protein, serving as opsonins for mesothelial cells. Moreover, mesothelial cells may play a role in clearing dead cell debris or microorganisms in the peritoneum during PD by phagocytosis. AIM may, thus, be involved in maintenance of a healthy peritoneal cavity by enhancement of phagocytosis by mesothelial cells as well as macrophages. In our human study we observed that low concentrations of AIM seem to be a risk factor for the occurrence of peritonitis (Supplementary Figure 8). AIM may bind to the surface of Gram-positive and Gram-negative bacteria, as well as to fungi^38–40^, resulting in their enhanced removal from the peritoneum by macrophages and mesothelial cells. Future studies using a larger cohort are needed to determine whether serum concentrations of AIM are a predictor of the occurrence of peritonitis in PD patients, and to study the effects of prevention of peritonitis by administration of AIM. In addition, whether or not fungal peritonitis is exaggerated in patients with low serum concentrations of AIM should be elucidated.

We observed that AIM was strongly detected in the necrotic area on day 14 (Fig. 4a). The fact that AIM mRNA expression in the peritoneum was upregulated in this study (Fig. 4c) indicates that local expression of AIM may be partly derived from local AIM production, as well as from circulating AIM. The mechanisms of binding of AIM to the targets, such as necrotic tissue, remain unclear. However, these phenomena were similar to those observed in the renal tubules, where AIM is bound to intra-tubular debris^29^. In recent studies, calcium-dependent phosphatidylserine exposure on the surface of necrotic cells has been reported to attract engulfing cells^31, 52^. Future studies are required to elucidate the precise mechanisms of AIM binding to dead cell debris. A limitation of this study is that the results and implications are only valid in a zymosan-induced peritonitis model, rather than an acute fungal infection model.

In summary, our observations show that AIM appears to be involved in the prevention and repair process of a PD-related fungal peritonitis model, and thus, can be the basis of development of new treatment strategies for PD-related fungal peritonitis.

## Concise Methods

Complete methods are shown in the Supplementary Methods section.

### Patient profiles and demographic data

The present study was performed in accordance with the ethical guidelines of the 1975 Declaration of Helsinki and was approved by the Ethics Committee for Human Research of the Faculty of Medicine, Nagoya University (Approval number #2013–0275). Informed consent in writing was obtained from all patients. All patients who were treated by PD at Nagoya University hospital from 2010–2013 were included in this cohort. No patients were excluded. A total of 59 serum samples of the patients on PD obtained at peritoneal equilibration tests (PET) were used for evaluations of peritoneal transport rate. All patients were free from peritonitis for more than 1 month before inclusion in the study. CAPD peritonitis was defined by the presence of a leukocyte count of 100 cells/mL or more in PD effluent, of which 50% or more were polymorphonuclear neutrophils^53^. All cases of peritonitis were bacterial in origin, and 21% were culture negative. There were no cases of fungal peritonitis and of development of EPS.

### Animal model and experimental design

Animal experiments were performed in accordance with the Animal Experimentation Guidelines of Nagoya University Graduate School of Medicine (Nagoya, Japan), and were approved by the Animal Experimentation Committee of Nagoya University (Approval #25378 and DNA#14–50). *AIM* ^−/−^ mice^34^ had been backcrossed to C57BL/6 (B6) for 13 generations before they were used in these experiments. In the present study, we used a zymosan-induced peritonitis model induced by five daily intra-peritoneal injections of 2 mg zymosan (Sigma-Aldrich, St. Louis, MO) diluted with 2 ml of saline in mice prepared by mechanical scraping of the right side of the parietal peritoneum, as we have previously described in detail (Supplementary Figure 1)^1, 41–43^. First, in order to address whether AIM is involved in the progression of zymosan-induced peritonitis models, we compared *AIM* ^+/+^ and *AIM* ^−/−^ mice (Supplementary Figure 1a). Next, we investigated whether treatment with rAIM (200 μg/ mouse) can ameliorate zymosan-induced peritonitis in *AIM* ^−/−^ mice (Supplementary Figure 1b).

### Histology and immunohistochemistry

Immunostaining for CD11b (1:50 dilution), AIM (1:4000), F4/80 (1:200) and cytokeratin was conducted using buffered formalin-fixed tissues^5, 54, 55^. Immunostaining for iNOS (1:100), CD4 (1:100), CD8 (1:200), CD11c (1:50), CD206 (1:200), F4/80, Ly6B2 (1:100), C3 (1:100) and C5b-9 (1:600) was conducted on 4-μm cryostat sections, as described previously^3, 4, 55^. The list of antibodies used in this study is shown in Supplementary Table 3. Immunostaining was observed using a Zeiss Z1 image microscope and Axiovision Windows software version 4.4 (Carl Zeiss, Oberkochen, Germany).

### RNA isolation from tissues and cultured cells, and quantitative PCR analysis

RNA isolation and synthesis of first-strand cDNA were conducted as described previously^3, 56^. Real-time polymerase chain reaction analysis was performed as described previously^3, 55^. A list of the TaqMan Gene Expression Assays (Applied Biosystems Inc., Foster City, CA) used for quantitative PCR experiments is shown in Supplementary Table 4.

### Western blotting of human serum AIM

Western blotting was performed as described previously^29^. Total AIM concentrations in the serum of PD patients were assessed by reducing immunoblot signals.

### Purification of rAIM

We generated and purified murine AIM (Cd51) as we described previously^29, 37^.

### Collection of cells derived from the peritoneum of zymosan model mice on day 7

On day 7, the peritoneums of each zymosan model mice were resected and cut into small pieces 1–2 mm in size. The pieces were transferred into gentleMACS^TM^ C tubes (Miltenyi Biotec, Tokyo, Japan) containing 5 ml Hank’s Balanced Salt Solution (HBSS, ThermoFisher Scientific, Waltham, MA), 5 mg collagenase type I (Worthington Biochemical, Lakewood, NJ) and 50 μl DNase I (40 U/ml, Sigma-Aldrich). After incubation for 20 min at 37 °C under continuous agitation, cells were obtained by gentleMACS^TM^ Dissociator (Miltenyi Biotec) using the gentleMACS^TM^ Program.

### Culture of mesothelial cells from AIM^+/+^ and AIM^−/−^ mice

Mouse parietal peritoneal mesothelial cells were obtained by digestion of parietal peritoneum from *AIM* ^+/+^ and *AIM* ^−/−^ mice as described previously^57^. In order to purify the mesothelial cells during assay of phagocytosis, CD11b positive cells were depleted using CD11b MicroBeads (Miltenyi Biotec) according to the manufacturer’s instructions.

### Bone marrow derived macrophage isolation and polarization

Preparation of M1- and M2a-like macrophages was performed according to previously established methods^58^. Briefly, bone marrow cells were harvested from the femur and tibia of *AIM* ^+/+^ and *AIM* ^−/−^ mice, injected into ice-cold RPMI 1640 medium (Sigma-Aldrich), and filtered through a 70-μm nylon mesh. One μl of LPS (Sigma-Aldrich, 4 mg/ml) and 1.5 μl of IFN-γ (20 ng/ml; Cell Signaling Technology, Inc., Danvers, MA) or 2 μl of IL-4 (20 ng/ml; Cell Signaling Technology, Inc.) and IL-13 (20 ng/ml; Cell Signaling Technology, Inc.) were used to transform the phenotype to M1 or M2a character, respectively.

### Preparation of dead cell debris using mesothelial cells (Met-5A) coated with AIM

Human mesothelial cell-line (Met-5A) cells, which were purchased from American Type Culture Collection (Manassas, VA), were cultured as described previously^3, 59^. Met-5A cells were heat-killed by incubation at 65 °C for 20 min in PBS, and labeled with FVD520, −660 or −780 (eBioscience) for 30 min at 4 °C. For coating of the surface of the cell debris with AIM, labeled dead cell debris was divided into two groups, and was incubated with serum-free culture medium with or without rAIM at a concentration of 50 μg/ml at 37 °C for 1 h, as described previously^29^.

### Phagocytosis assay using the peritoneal cells derived from the peritoneum of zymosan model mice

Peritoneal cells (10^6^ cells/sample) derived from zymosan model mice on day 7 were mixed with dead cell debris labeled with FVD780 with or without AIM coating, in serum free DMEM/F-12 medium supplemented with 5 μg/ml insulin, 5 μg/ml transferrin and 5 ng/ml selenous acid at 37 °C for 10 and 30 min. Then, the cells were washed twice with ice-cold MACS buffer. Thereafter, the cells were incubated with Allophycocyanin (APC) labeled anti-mouse Mac-1(CD11b) antibody (eBioscience), Phycoerythrin (PE)-labeled anti-mouse Ly6G (Gr-1, eBioscience) and fluorescein isothiocyanate (FITC)-labeled anti-mouse F4/80 (eBioscience), and were re-suspended with MACS buffer containing 4′,6-diamidino-2-phenylindole (DAPI, Sigma-Aldrich). The cells were then subjected to flow cytometry (BD LSRII, BD Biosciences, San Jose, CA). The proportion of engulfment of FVD780-positive cell debris within DAPI^−^Mac-1^+^F4/80^+^Gr-1^−^ cells and DAPI^−^Mac-1^+^F4/80^−^Gr-1^+^ cells was assessed.

### Phagocytosis assay using cultured M1- and M2a-like macrophages or mesothelial cells

Cultured M1- or M2a-like macrophages and mesothelial cells (10^6^ cells/sample) were stained by CellTracker™ Green CMFDA Dye (5-Chloromethylfluorescein Diacetate, Thermo Fisher Scientific) at 10 μM, and mixed with debris labeled by FVD660 (eBioscience) with or without rAIM coating for 10, 30 or 90 min at 37 °C. After incubation, the cells were harvested, washed twice with ice-cold MACS buffer, resuspended in MACS buffer containing 7-Amino-actinomycin D (7AAD, BD Biosciences) to identify the living cells, and were then subjected to BD FACS Canto II (BD Biosciences). The proportion of engulfment of FVD660 positive dead cell debris in 7AAD negative mesothelial cells or macrophages was assessed. We also observed engulfment of cell debris by macrophages or mesothelial cells using an Incubator Fluorescence Microscope (LCV110, Olympus, Tokyo, Japan).

### Confocal microscopy studies

In addition, we observed the above phenomena using confocal microscopy (TiEA1R, NIKON INSTECH Co. Ltd., Tokyo Japan) under the conditions of with or without AIM coating. Debris was stained with FVD520 (eBioscience). The peritoneal cells derived from the zymosan model mice were stained with APC anti-mouse F4/80 antibody (BioLegend, San Diego, CA) and incubated with Anti-APC microbeads (Miltenyi Biotec). F4/80 positive cells were separated using a MACS LS column (Miltenyi Biotec) stained with CellTracker™ Red CMPTX Dye (10 µM) (Thermo Fisher Scientific).

### Statistical Analyses

Variables with a normal distribution are described as mean values ± SD, and asymmetrical distributions are presented as median and interquartile range. Categorical variables are given as numbers and percentages. To assess the differences between two groups, Student’s *t* test or the Mann-Whitney *U* test was used. Fisher’s exact test was employed when variables were categorical. Comparisons among groups were performed by one-way analysis of variance (ANOVA) followed by Dunnett’s or Kruskal-Wallis multiple comparison tests. Differences were considered to be statistically significant if *P* value was <0.05. All analyses were conducted by SPSS software (SPSS, Chicago, IL).

## Electronic supplementary material

Supplementary Information

Supplementary video 1

Supplementary video 2

Supplementary video 3
