# Supplementary material for: Apoptosis inhibitor of macrophage ameliorates fungus-induced peritoneal injury model in mice
Source: Sci Rep. 2017 Jul 25;7:6450. doi: 10.1038/s41598-017-06824-6 (PMC5527077; doi:10.1038/s41598-017-06824-6)
Supplement: Supplementary file 1 — Supplementary Information [file 41598_2017_6824_MOESM1_ESM.pdf]

Supplementary Information

Apoptosis inhibitor of macrophage ameliorates fungus-induced peritoneal injury model in mice

Takako Tomita, Satoko Arai, Kento Kitada, Masashi Mizuno, Yasuhiro Suzuki, Fumiko Sakata, Daisuke Nakano, Emiri Hiramoto, Yoshifumi Takei, Shoichi Maruyama, Akira Nishiyama, Seiichi Matsuo, Toru Miyazaki and Yasuhiko Ito

1. **Supplementary Figures 1-8**
2. **Supplementary Tables 1-4**
3. **Supplementary Methods**
4. **Supplementary Videos 1-3**
   (Please see the attached video files)

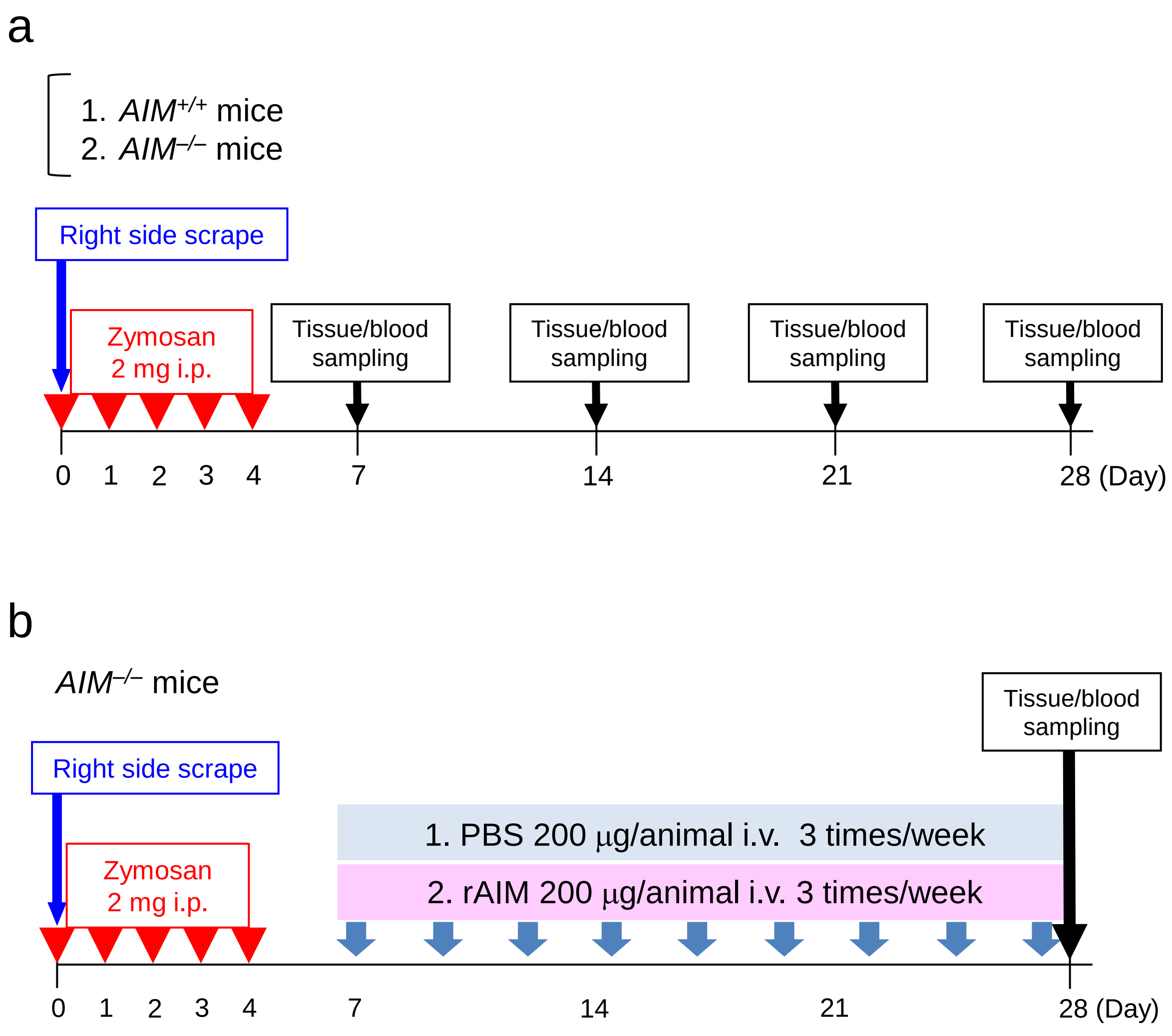

Supplementary Figure 1. Animal experimental design.

a: Zymosan-induced peritonitis models were created in *AIM*$^{+/+}$ and *AIM*$^{-/-}$ mice. They were sacrificed on days 0, 7, 14, 21 (*n*=6 in each group) and 28 (*n*=14).
b: Zymosan model *AIM*$^{-/-}$ mice were treated with rAIM or PBS.
Two hundred μg of rAIM was administered intravenously three times per week from day 7 to 28, following which the animals were sacrificed and assessed (*n*=11 in each group).

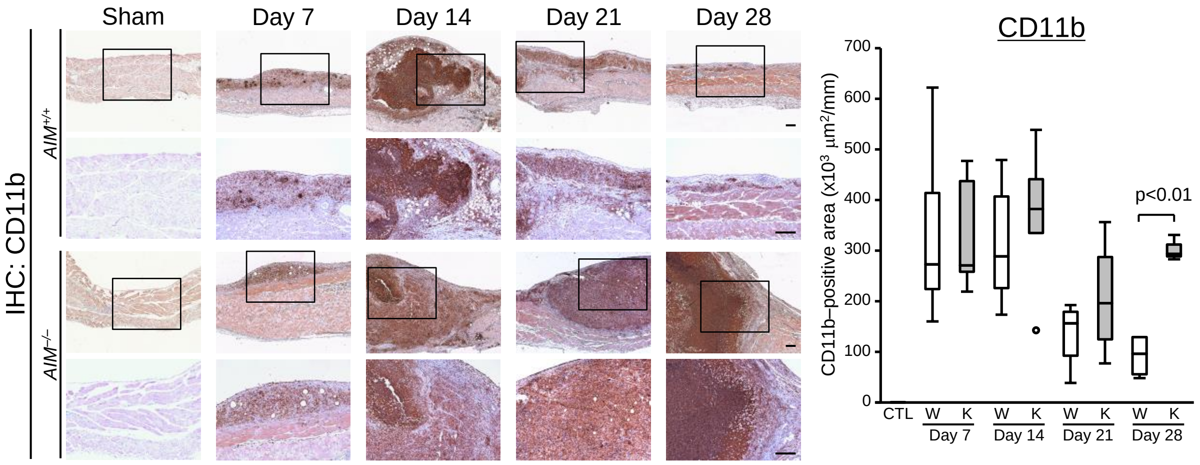

Supplementary Figure 2. Morphological changes in the zymosan-induced peritonitis model.

Expression of CD11b positive cells in the zymosan-induced peritonitis model. Infiltration of CD11b positive cells continued up to 4 weeks in AIM-deficient mice, while it decreased 3 weeks after disease induction in the wild mice. Generally, the CD11b antigen is reported to be strongly expressed in monocytes/macrophages, and expressed in granulocytes and NK cells. F4/80 is expressed in all types of macrophages (Reference a).
Left figures: The second and fourth rows show higher magnifications of the images in the boxed areas in the corresponding images in the first and third rows, respectively.
Right graphs: CD11b positive areas were assessed by morphometry and were expressed as $\times 10^3$ $\mu m^2/\mu m$ surface length.
W: *AIM*$^{+/+}$ mice; K: *AIM*$^{-/-}$ mice; CTL: control normal *AIM*$^{+/+}$ mice, Scale bars, 200 μm.
n=6 for each group.

Reference a. Gabrilovich DI, Ostrand-Rosenberg S, Bronte V.
Coordinated regulation of myeloid cells by tumours. *Nat Rev Immunol.* **12**, 253-68 (2012).

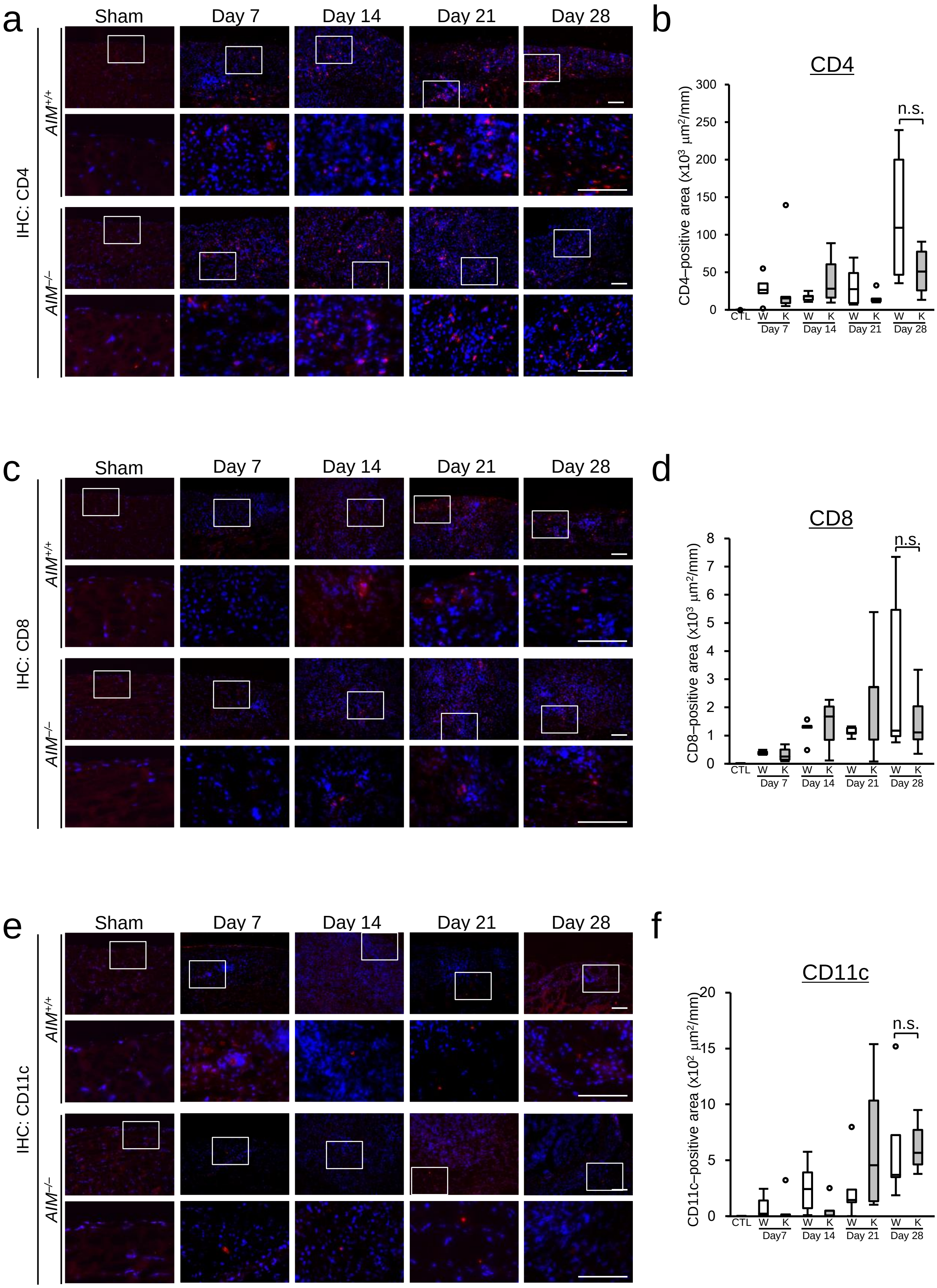

Supplementary Figure 3

Supplementary Figure 3. Expression of surface markers of infiltrating cells in the zymosan-induced peritonitis model: Immunostaining of the peritoneal wall for CD4 (a, b), CD8 (c, d) and CD11c (e, f).

On day 28, there were no significant differences in the expression of CD4 (**a, b**), CD8 (**c, d**) and CD11c (**e, f**) positive cells between *AIM*$^{-/-}$ and *AIM*$^{+/+}$ mice. The nuclei were counterstained with DAPI (blue color). The second and fourth rows of images show higher magnifications of the white boxed areas in the corresponding images in the first and third rows, respectively.
W: *AIM*$^{+/+}$ mice, K: *AIM*$^{-/-}$ mice, Scale bars, 100 μm. *n*=6 for each group.

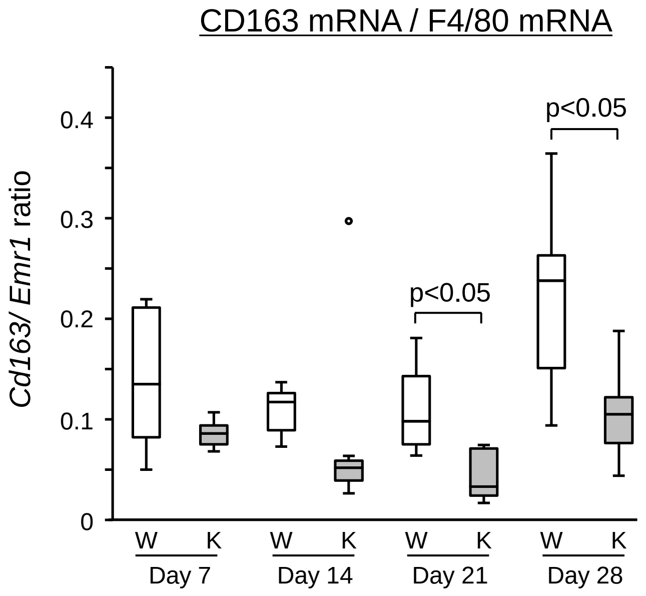

Supplementary Figure 4. Expression of inflammatory markers in the zymosan-induced peritonitis model, as assessed by quantitative real-time PCR.

Cd163 (CD163) mRNA/*Emr1* (F4/80) mRNA.
W: *AIM*$^{+/+}$ mice; K: *AIM*$^{-/-}$ mice, CTL: control normal *AIM*$^{+/+}$ mice. *n*=6 for each group.

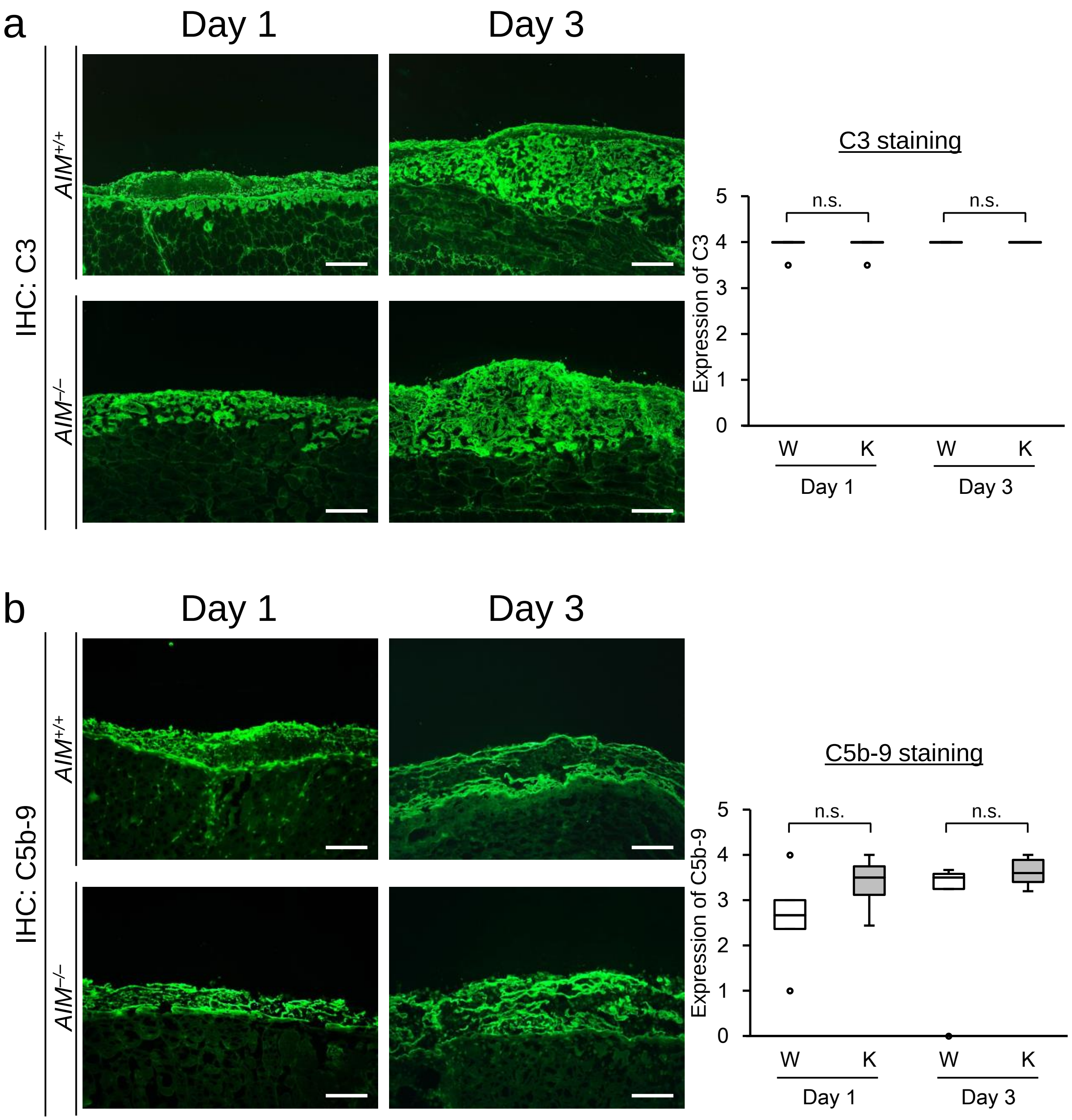

Supplementary Figure 5. Activation of complement in the early phase of the zymosan-induced peritonitis model was similar between *AIM*$^{+/+}$ and *AIM*$^{-/-}$ mice.

a: C3 deposition in the peritoneal wall in *AIM*$^{+/+}$ and *AIM*$^{-/-}$ mice.
b: Membrane attack complex (C5b-9) deposition in the peritoneal wall in AIM$^{+/+}$ and *AIM*$^{-/-}$ mice.
W: *AIM*$^{+/+}$ mice, K: *AIM*$^{-/-}$ mice, Scale bars, 100 μm.
n=6 for each group.

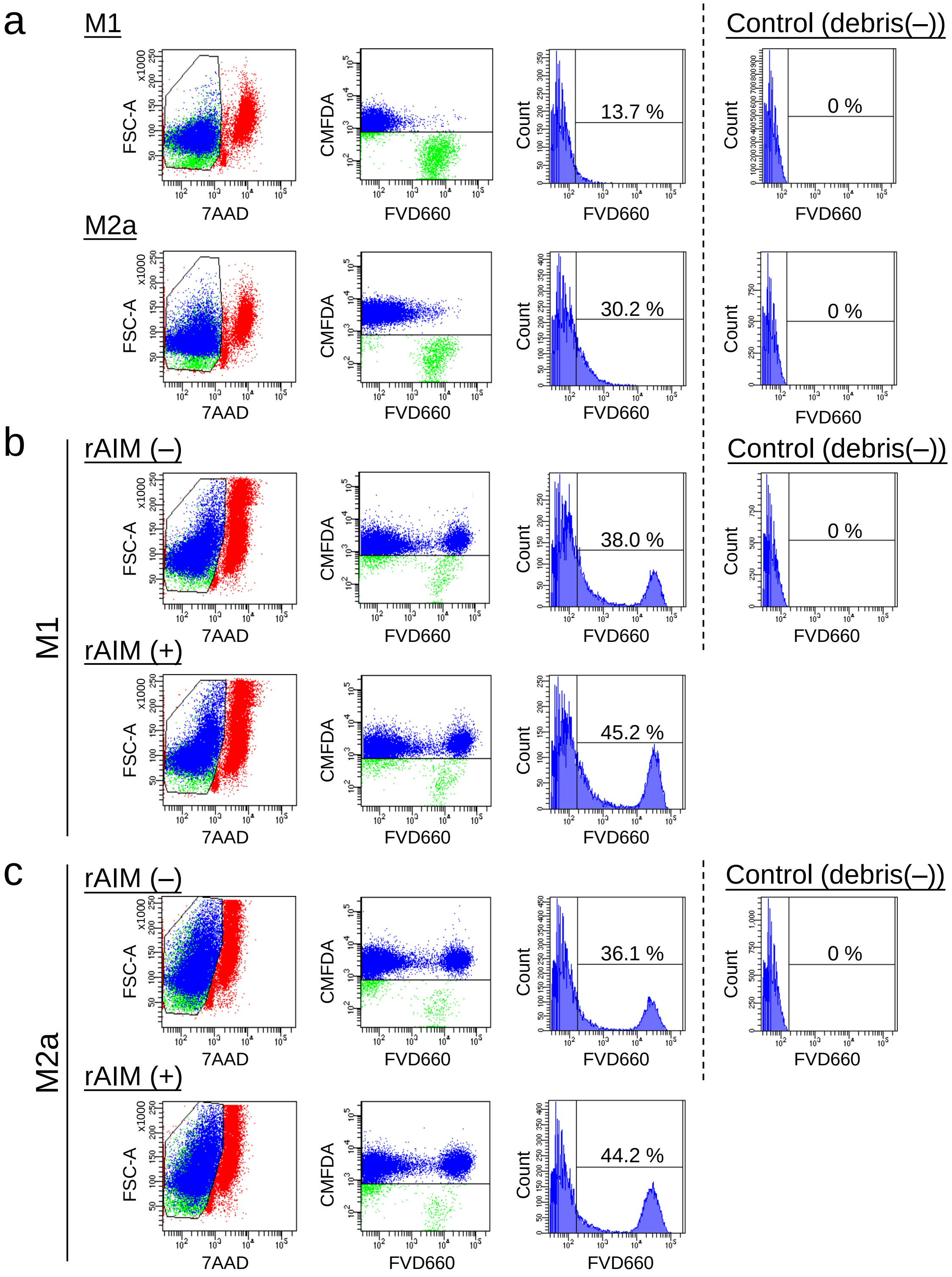

Supplementary Figure 6

Supplementary Figure 6. M2a-like macrophages had a greater engulfing capacity than M1-like macrophages (a). Coating with AIM enhanced debris engulfment by both M1- (b) and M2a- (c) like macrophages.

This is the supplementary information for **Figures 6b and- c**. Cultured M1- and M2a-like macrophages were stained with CellTracker™ Green CMFDA Dye, and mixed with debris labeled with FVD660 with or without rAIM coating (**b, c**). After incubation for 10 min (**a**) or 30 min (**b, c**), the cells were harvested, twice-washed with ice-cold MACS buffer, resuspended in MACS buffer containing 7-Amino-Actinomycin D (7AAD), and were then subjected to BD FACS Canto II. The proportion of engulfment of FVD660 positive dead cell debris by 7AAD negative macrophages was assessed. **a** to **c** indicate the flow cytometry using macrophages from *AIM*$^{-/-}$ mice.

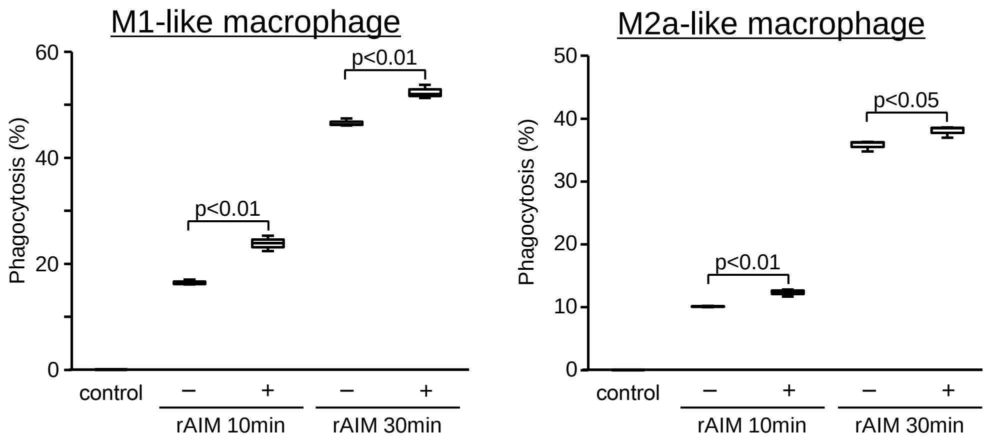

Supplementary Figure 7. Coating with AIM enhanced debris engulfment by M1- and M2a-like macrophages of *AIM*$^{+/+}$ mice.

The same experiments as for **Figure 6c** and **Supplementary Figures 6b and- c** were performed using *AIM*$^{+/+}$ mice. These findings indicate that supplementation of AIM promotes phagocytosis of dead cell debris by macrophages from *AIM*$^{+/+}$ as well as *AIM*$^{-/-}$ mice.
n=3 dishes for each group.

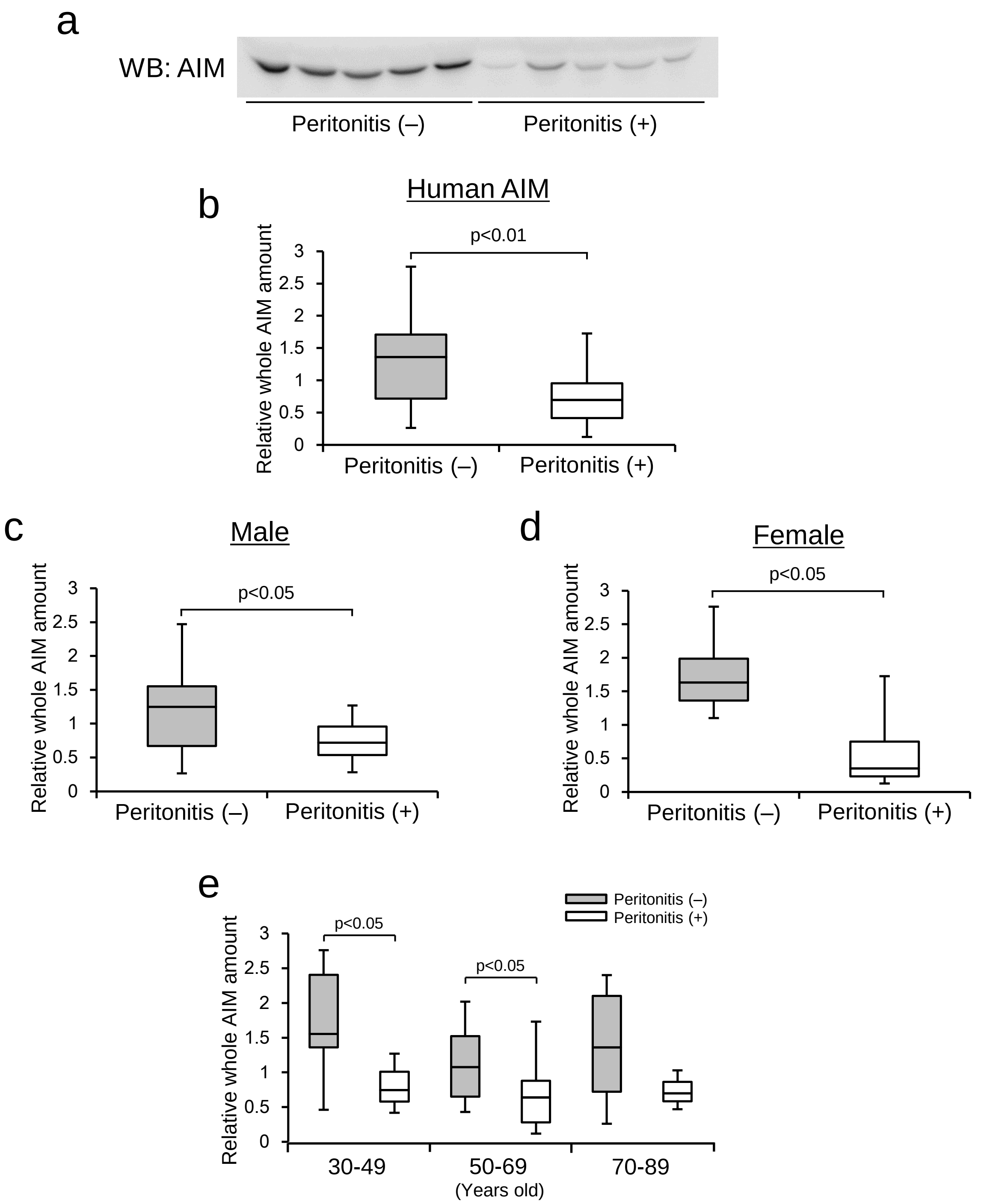

Supplementary Figure 8. Circulating AIM levels were lower in patients with a history of PD-related peritonitis than those without peritonitis.

a: Serum total AIM by Western blotting under reducing conditions,
b: Quantification of serum total AIM concentrations,
c-e: Serum total AIM classified according to gender (**c:** male, **d:** female) or age (**e**). Serum AIM was significantly lower in the PD patients who had previous experience of peritonitis than those who had not.
b: Peritonitis (+) (*n*=18), Peritonitis (−) (*n*=41),
c: Peritonitis (+) (*n*=12), Peritonitis (−) (*n*=30),
d: Peritonitis (+) (*n*=6), Peritonitis (−) (*n*=11),
e: 30-49 years old (*n*=16), 50-69 years old (*n*=30), 70-89 years old (*n*=13).

	Day7		Day14		Day21		Day28	
	AIM$^{+/+}$	*AIM*$^{-/-}$	*AIM*$^{+/+}$	*AIM*$^{-/-}$	*AIM*$^{+/+}$	*AIM*$^{-/-}$	*AIM*$^{+/+}$	*AIM*$^{-/-}$
Ly6B2	↑	↑	⬆	⬆	↓	↓	⬇	→
CD11b	↑	↑	⬆	⬆	↓	↓	⬇	→
F4/80	↑	↑	⬆	⬆	↓	→	⬇	↓
iNOS(M1)	↑	↑	⬆	⬆	↓	↓	⬇	↓
CD206(M2)	↑	↑	↓	↓	⬆	↓	⬆	→

Supplementary Table 1. Summary of the expression of inflammatory cells infiltration of the zymosan-induced peritonitis model in both *AIM*$^{+/+}$ and *AIM*$^{-/-}$ mice.

	Total (n = 59)	Peritonitis (–) (n = 41)	Peritonitis (+) (n = 18)	P-value	
Sex					
male	42(71%)	30(73%)	12(67%)	0.612	*
female	17(29%)	11(27%)	6(33%)		
Age	61.5±12.7	57.6±13.8	60.8±10.8	0.326	†
Primary disease of chronic kidney disease					
diabetes	19(32%)	13(32%)	6(33%)	0.902	*
Treatment period (month)	40.4±26.2	36.1±24.8	49.7±27.5	0.05	‡
APD	19(32%)	16(39%)	3(17%)	0.091	*
Serum Alb (g/dl)	3.33±0.44	3.38±0.43	3.25±0.45	0.261	‡
Protein concentration in PD effluent (μg/ml)	1756±556	1786±568	1687±538	0.598	‡
D/P Cr	0.66±0.10	0.66±0.10	0.65±0.09	0.95	†
Clearance study					
Urine volume(mL/day)	936±877	976±880	852±887	0.593	‡
Dialysate drain volume(mL/day)	7579±3584	7693±3950	7331±2708	0.911	‡
Total Kt/V	1.85±0.43	1.82±0.45	1.93±0.38	0.404	‡
Renal Kt/V	0.51±0.56	0.52±0.54	0.49±0.60	0.744	‡
Peritoneal Kt/V	1.34±0.54	1.30±0.57	1.44±0.47	0.221	‡

* n (%), Fisher's exact test † mean±SD, Student's *t* test ‡ median (IQR), Mann-Whitney *U* test

APD: Automated Peritoneal Dialysis, Alb: albumin, D/P: dialysate / plasma,
Kt/V: amount of dialysis delivered: K = clearance of urea, t = time on dialysis, V = estimated total body water

Supplementary Table 2. Demographic data of the cases evaluated for serum levels of AIM.

Antibody	Company
Anti-CD11b antibody	Abcam, Cambridge, UK
rat anti-mouse F4/80 antibody	AbD Serotec, Oxfordshire, UK
rabbit anti-iNOS antibody	Abcam, Cambridge, UK
rat anti-mouse CD206 antibody	AbD Serotec, Oxfordshire, UK
rat anti-mouse Ly6B2 antibody	AbD Serotec, Oxfordshire, UK
american hamster anti-CD11c antibody	Abcam, Cambridge, UK
rat anti-CD4 antibody	Abcam, Cambridge, UK
rat anti-CD8 antibody	Abcam, Cambridge, UK
FITC-labeled goat anti-mouse C3	Cappel, Solon, OH
rabbit anti-C5b-9 antibody	provided from prof. Morgan BP (Cardiff University, Cardiff, UK)
rabbit polyclonal anti-AIM antibody	generated by prof.T Miyazaki (Tokyo University, Tokyo, Japan)
monoclonal anti-pan cytokeratin	Sigma-Aldrich, St. Louis, MO
FITC-labbeled goat anti-rat IgG F(ab')2	AbD Serotec, Oxfordshire, UK
FITC-labeled goat anti-rabbit IgG(H+L)	Life Technologies, Carlsbad, CA
FITC-labeled rabbit anti-rat IgG(H+L)	Jackson Immuno Research, West Grove, PA
Rhodamine-labeled goat anti-rabbit IgG	Merck Millipore, Darmstadt, DE
Alexa555-labeled gout anti-rat IgG(H+L)	Cell Signaling Technology, Inc, Denvers, MA
Alexa568-labeled goat anti-hamster IgG(H+L)	Life Technologies, Carlsbad, CA

Supplementary Table 3. List of antibodies used for immunohistochemistry in this study.

	Assay identification number
Mouse *Il6* (IL-6)	Mm00446190_m1
Mouse *Tnf* (TNF-α)	Mm00443258_m1
Mouse *Nos2* (iNOS)	Mm00440502_m1
Mouse *Cd206* (CD206)	Mm01329362_m1
Mouse *Cd163* (CD163)	Mm00474091_m1
Mouse *Emr1* (F4/80)	Mm00802529_m1
Mouse *Cd5l* (AIM)	Mm00437566_g1
18S ribosomal RNA	4319413E

Supplementary Table 4. List of primers used for real-time PCR (TaqMan Gene Expression Assays) in this study.

Supplementary Methods

Patient profiles and demographic data

The present study was performed in accordance with the ethical guidelines of the 1975 Declaration of Helsinki and was approved by the Ethics Committee for Human Research of the Faculty of Medicine, Nagoya University (Approval number #2013-0275). Informed consent in writing was obtained from all patients. All patients who were treated by PD at Nagoya University hospital from 2010-2013 were included in this cohort. No patients were excluded. All patients used germicidal exchange devices for PD (UV flash auto, Baxter Healthcare, Tokyo, Japan), and the occurrence rate of peritonitis in this cohort was about 0.1 $patient^{-1} \times year^{-1}$. A total of 59 serum samples of the patients on PD obtained at peritoneal equilibration tests (PET) were used for evaluations of peritoneal transport rate. The mean age of all patients was 61.5±12.7 years, and the mean duration of PD treatment was 40.4±26.2 months. Diabetic nephropathy was the primary cause of end-stage renal disease in 19 PD patients (32%). All patients were free from peritonitis for more than 1 month before inclusion in the study. Peritoneal transport was assessed at the time of PET as the ratio of Dialysate/Plasma creatinine (D/P Cr), and its average value was 0.66±0.10. CAPD peritonitis was defined by the presence of a leukocyte count of 100 cells/mL or more in PD effluent, of which 50% or more were polymorphonuclear neutrophils (S-1). All cases of peritonitis were bacterial in origin, and 21% were culture negative. There were no cases of fungal peritonitis and of development of EPS.

Animal model and experimental design

Animal experiments were performed in accordance with the Animal Experimentation Guidelines of Nagoya University Graduate School of Medicine (Nagoya, Japan), and

were approved by the Animal Experimentation Committee of Nagoya University (Approval # 25378 and DNA#14-50). *AIM*$^{-/-}$ mice (S-2) had been backcrossed to C57BL/6 (B6) for 13 generations before they were used in these experiments. Eight to ten-week-old male *AIM*$^{+/+}$ and *AIM*$^{-/-}$ mice weighing 20-25 g were used throughout the study. Animals were maintained under specific-pathogen-free conditions and had free access to food and water. In the present study, we used a zymosan-induced peritonitis model induced by five daily intra-peritoneal injections of 2 mg zymosan (Sigma-Aldrich, St. Louis, MO) diluted with 2 ml of saline in mice prepared by mechanical scraping of the right side of the parietal peritoneum, as we have previously described in detail (**Supplementary Figure 1**) (S-3-6). First, in order to address whether AIM is involved in the progression of zymosan-induced peritonitis models, we compared *AIM*$^{+/+}$ and *AIM*$^{-/-}$ mice. Mice in both groups were sacrificed on days 7, 14, 21 or 28 under anesthesia with Isoflurane (Wako Pure Chemical Industries, Osaka, Japan). Blood was drawn and parietal peritoneal walls were harvested and processed for routine histology, immunohistochemistry and total RNA isolation (**Supplementary Figure 1a**). Next, we investigated whether treatment with rAIM (200μg/ mouse) can ameliorate zymosan-induced peritonitis in *AIM*$^{-/-}$ mice (**Supplementary Figure 1b**). In zymosan models of *AIM*$^{-/-}$ mice, 200 μg/ 20 g body weight of rAIM was intravenously administered 3 times per week from day 7 to day 28, and the mice were sacrificed on day 28. Blood samples and peritoneal wall samples were obtained from them.

Sample processing and analysis

One part of each tissue sample was fixed with 10% buffered formalin overnight, following which the samples were routinely processed and embedded in paraffin, and the cut sections were stained with hematoxylin and eosin (HE) and Masson’s trichrome

for light microscopy and were used for immunohistochemistry (IHC). A second fragment was snap-frozen in liquid nitrogen, and the tissues were cut with a cryostat and used for IHC. A third fragment of each tissue sample was immersed in RNA*later* (Ambion, Austin, TX) to isolate RNA. For preservation of the integrity and stability of total RNA, all the procedures were conducted at 4°C under sterile conditions.

Histology and immunohistochemistry

Immunostaining for CD11b (1:50 dilution), AIM (1:4000), F4/80 (1:200) and cytokeratin was conducted using buffered formalin-fixed tissues (S-7-9). Immunostaining for iNOS (1:100), CD4 (1:100), CD8 (1:200), CD11c (1:50), CD206 (1:200), F4/80, Ly6B2 (1:100), C3 (1:100) and C5b-9 (1:600) was conducted on 4-μm cryostat sections, as described previously (S-9-11). The list of antibodies used in this study is shown in **Supplementary Table** 3.

Morphological analysis

Immunostaining was observed using a Zeiss Z1 image microscope and Axiovision Windows software version 4.4 (Carl Zeiss, Oberkochen, Germany). Positive areas for F4/80, CD4, CD8, CD11b, CD11c, CD206, Ly6B and iNOS were quantified by MetaMorph 6.3 image software (Universal Imaging, West Chester, PA) (S-10, 11). The average of at least 10 random 750 × 500 μm areas was presented. Positive areas were assessed by morphometry and were expressed as $\times 10^3$ μm^2/mm surface length. Expression of C3 and C5b-9 was semi-quantitatively classified into 5 groups according to the intensity of the positive staining: (0) no; (1) weak; (2) mild; (3) moderate; and (4) pronounced staining (S-3). The average of the scores was calculated and defined as the C3 or C5b-9 score.

RNA isolation from tissues and cultured cells, and quantitative PCR analysis

RNA isolation and synthesis of first-strand cDNA were conducted as described previously (S-10, 12). One μg of total RNA from each peritoneum sample was then reverse transcribed. Real-time polymerase chain reaction analysis was performed using an Applied Biosystems Prism 7500HT sequence detection system with TaqMan gene expression assays, as described previously (S-9, 10). A list of the TaqMan Gene Expression Assays (Applied Biosystems Inc., Foster City, CA) used in this experiment is shown in **Supplementary Table** 4. We used 18S ribosomal RNA as an endogenous control.

Western blotting of human serum AIM

Western blotting was performed as described previously (S-13). Briefly, 1 μL of mouse or human serum was dissolved in NuPAGE 12% Bis-Tris Gel (Life Technologies, Carlsbad, CA) under reducing conditions, and proteins were transferred to Immobilon-P Membrane (Merck Millipore, Billerica, MA). The membranes were blocked with 5% skim milk in PBS-Tween-20 (Sigma-Aldrich) for 1 h at room temperature, then incubated overnight at 4°C with rabbit anti-AIM antibody diluted in blocking buffer and probed with goat anti-rabbit-HRP (Jackson ImmunoResearch, West Grove, PA) diluted with blocking buffer for 120 min at room temperature. Three times of 10 min washes between steps were performed with PBS-T. Bound antibody was detected with ImmunoStar LD (Wako).

Purification of rAIM

We generated and purified murine AIM (Cd51) as we described previously (S-13, 14). Briefly, the recombinant plasmids encoding musculus Cd5l were transfected to Chinese hamster ovary (CHO)-3E7 cells. Culture supernatants were collected and purified by rat anti-murine AIM monoclonal antibody-conjugated Protein G Sepharose (GE Healthcare,

Uppsala, Sweden). Bound protein was eluted with 0.1 M Glycine-HCl (pH 3.0) and neutralized with 1 M Tris-HCl (pH 8.5). The protein was concentrated by Amicon Ultra filter concentrators (Merck Millipore). Endotoxin concentrations were measured by the chromogenic LAL endotoxin detection system (Genscript, Piscataway, NJ). The purified protein was analyzed by Western blotting and was detected as 36 KD.

Collection of cells derived from the peritoneum of Zymosan model mice on day 7

On day 7, the peritoneums of each zymosan model mice were resected and cut into small pieces 1-2 mm in size. The pieces were transferred into gentleMACS™ C tubes (Miltenyi Biotec, Tokyo, Japan) containing 5 ml Hank's Balanced Salt Solution (HBSS, ThermoFisher Scientific, Waltham, MA), 5 mg collagenase type I (Worthington Biochemical, Lakewood, NJ) and 50 μl DNase I (40U/ml, Sigma-Aldrich). After incubation for 20 min at 37°C under continuous agitation, cells were obtained by gentleMACS™ Dissociator (Miltenyi Biotec) using the gentleMACS™ Program.

Culture of mesothelial cells from AIM$^{+/+}$ and AIM$^{-/-}$ mice

Mouse parietal peritoneal mesothelial cells were obtained by digestion of parietal peritoneum from *AIM*$^{+/+}$ and *AIM*$^{-/-}$ mice as described previously (S-15). Briefly, the peritoneal membrane was digested with a 0.125% trypsin solution for 15 min and occasionally agitated at 37°C. Cells were cultured in DMEM/F-12 media supplemented with 20% fetal calf serum, 50 U/ml penicillin, 50 μg/ml streptomycin, and 1% Biogro-2 (Biological Industries Israel Beit Haemek Ltd., Israel). In order to purify the mesothelial cells during assay of phagocytosis, CD11b positive cells were depleted using CD11b MicroBeads (Miltenyi Biotec) according to the manufacturer’s instructions. The purity of mesothelial cells was more than 98%, as verified by flow cytometric analysis and immuno-histochemistry for cytokeratin, CD11b and α-smooth muscle antigen.

Bone marrow derived macrophage isolation and polarization

Preparation of M1- and M2a-like macrophages was performed according to previously established methods (S-16). Briefly, bone marrow cells were harvested from the femur and tibia of *AIM*$^{+/+}$ and *AIM*$^{-/-}$ mice, injected into ice-cold RPMI 1640 medium (Sigma-Aldrich) using sterile syringes, and filtered through a 70-μm nylon mesh. The cell suspension was put onto the density gradient cell separation medium Histopaque (Sigma-Aldrich) and centrifuged for 30 min at 400×*g* and 25°C. The cells were collected from the interfacial layer of Histopaque, washed, and then sowed into the 10 cm dish with 10 ml of macrophage culture medium (MCM; RPMI 1640 medium-FBS, containing 20% v/v L929 cell-conditioned medium as a source of M-CSF) (S-16). On day 3, floating cells were collected from the culture supernatant and sowed in a 5×10^6-10×10^6/dish with MCM. On day 7, 1 μl of LPS (Sigma-Aldrich, 4 mg/ml) and 1.5 μl of IFN-γ (20 ng/ml; Cell Signaling Technology, Inc., Danvers, MA) or 2 μl of IL-4 (20 ng/ml; Cell Signaling Technology, Inc.) and IL-13 (20 ng/ml; Cell Signaling Technology, Inc.) were added to transform the phenotype to M1 or M2a character, respectively. On day 9, these cells were used for the experiments. Characterization of these cells was assessed by flow cytometry (BD FACS Canto II, BD Biosciences, Tokyo, Japan), which showed higher expression of CD86 for M1-like macrophages and of CD206 for M2a-like macrophages.

Preparation of dead cell debris using mesothelial cells (Met-5A) coated with AIM

Human mesothelial cell-line (Met-5A) cells, which were purchased from American Type Culture Collection (Manassas, VA), were cultured as described previously (S-10,17). Met-5A cells were heat-killed by incubation at 65 °C for 20 min in PBS, and labeled with FVD520, -660 or -780 (eBioscience) for 30 min at 4 °C. For coating of the

surface of the cell debris with AIM, labeled dead cell debris was divided into two groups, and was incubated with serum-free culture medium with or without rAIM at a concentration of 50 μg/ml at 37 °C for 1 h, as described previously (S-13).

Phagocytosis assay using the peritoneal cells derived from the peritoneum of zymosan model mice

Peritoneal cells (10^6 cells/sample) derived from zymosan model mice on day 7 were mixed with dead cell debris labeled with FVD780 with or without AIM coating, in serum free DMEM/F-12 medium supplemented with 5 μg/ml insulin, 5 μg/ml transferrin and 5 ng/ml selenous acid at 37°C for 10 and 30 min. Then, the cells were washed twice with ice-cold MACS buffer. Thereafter, the cells were incubated with Allophycocyanin (APC) labeled anti-mouse Mac-1(CD11b) antibody (eBioscience), Phycoerythrin (PE)-labeled anti-mouse Ly6G (Gr-1, eBioscience) and fluorescein isothiocyanate (FITC)-labeled anti-mouse F4/80 (eBioscience), and were re-suspended with MACS buffer containing 4',6-diamidino-2-phenylindole (DAPI, Sigma-Aldrich). The cells were then subjected to flow cytometry (BD LSRII, BD Biosciences, San Jose, CA). The proportion of engulfment of FVD780-positive cell debris within $DAPI^{-}Mac\text{-}1^{+}F4/80^{+}Gr\text{-}1^{-}$ cells and $DAPI^{-}Mac\text{-}1^{+}F4/80^{-}Gr\text{-}1^{+}$ cells was assessed.

Phagocytosis assay using cultured M1- and M2a-like macrophages or mesothelial cells

Cultured M1- or M2a-like macrophages and mesothelial cells (10^6 cells/sample) were stained by CellTracker™ Green CMFDA Dye (5-Chloromethylfluorescein Diacetate, Thermo Fisher Scientific) at 10 μM, and mixed with debris labeled by FVD660 (eBioscience) with or without AIM coating for 10, 30 or 90 min at 37 °C. After incubation, the cells were harvested, washed twice with ice-cold MACS buffer,

resuspended in MACS buffer containing 7-Amino-actinomycin D (7AAD, BD Biosciences) to identify the living cells, and were then subjected to BD FACS Canto II (BD Biosciences). The proportion of engulfment of FVD660 positive dead cell debris in 7AAD negative mesothelial cells or macrophages was assessed. We also observed engulfment of cell debris by macrophages or mesothelial cells using an Incubator Fluorescence Microscope (LCV110, Olympus, Tokyo, Japan).

Confocal microscopy studies

In addition, we observed the above phenomena using confocal microscopy (TiEA1R, NIKON INSTECH Co. Ltd., Tokyo Japan) under the conditions of with or without AIM coating. Debris was stained with FVD520 (eBioscience). The peritoneal cells derived from the zymosan model were incubated with CD16/CD32 (BD Biosciences) to prevent nonspecific binding, then stained with APC anti-mouse F4/80 antibody (BioLegend, San Diego, CA) and incubated with Anti-APC microbeads (Miltenyi Biotec). F4/80 positive cells were separated using a MACS LS column (Miltenyi Biotec) stained with CellTracker™ Red CMPTX Dye (10 µM) (Thermo Fisher Scientific).

Statistical Analyses

Variables with a normal distribution are described as mean values ± SD, and asymmetrical distributions are presented as median and interquartile range. Categorical variables are given as numbers and percentages. To assess the differences between two groups, Student's *t* test or the Mann-Whitney *U* test was used. Fisher's exact test was employed when variables were categorical. Comparisons among groups were performed by one-way analysis of variance (ANOVA) followed by Dunnett's or Kruskal-Wallis multiple comparison tests. Differences were considered to be statistically significant if *P* value was <0.05. All analyses were conducted by SPSS software (SPSS, Chicago, IL).

REFERENCES

S-1. Li PK, et al. ISPD Peritonitis Recommendations: 2016 Update on Prevention and Treatment. *Perit Dial Int.* **36**, 481-508 (2016).

S-2 Miyazaki T, et al. Increased susceptibility of thymocytes to apoptosis in mice lacking AIM, a novel murine macrophage-derived soluble factor belonging to the scavenger receptor cysteine-rich domain superfamily. *J Exp Med.* **189**, 413–22 (1999).

S-3. Mizuno M, et al. Zymosan, but not lipopolysaccharide, triggers severe and progressive peritoneal injury accompanied by complement activation in a rat peritonitis model. *J immunol.* **183**, 1403–12 (2009).

S-4. Mizuno M, Ito Y. Rat Models of Acute and/or Chronic Peritoneal Injuries Including Peritoneal Fibrosis and Peritoneal Dialysis Complications. *Methods Mol Biol.* **1397**, 35–43 (2016).

S-5. Mizuno M, et al. Membrane complement regulators protect against fibrin exudation increases in a severe peritoneal inflammation model in rats. *Am J Physiol Renal Physiol.* **302**, F1245–51 (2012).

S-6. Ito Y, et al. Peritonitis-induced peritoneal injury models for research in peritoneal dialysis review of infectious and non-infectious models. *Renal Replacement Therapy.* **3**,16; 10.1186/s41100-017-0100-4 (2017).

S-7. Nishimura H, et al. Mineralocorticoid receptor blockade ameliorates peritoneal fibrosis in new rat peritonitis model. *Am J Physiol Renal Physiol.* **294**, F1084–93 (2008).

S-8. Sakamoto I, et al. Lymphatic vessels develop during tubulointerstitial fibrosis. *Kidney Int.* **75**, 828–38 (2009).

S-9. Suzuki Y, et al. Transforming growth factor-β induces vascular endothelial growth factor-C expression leading to lymphangiogenesis in rat unilateral ureteral obstruction. *Kidney Int.* **81**, 865–79 (2012).

S-10. Kinashi H, et al. TGF-β1 promotes lymphangiogenesis during peritoneal fibrosis. *J Am Soc Nephrol.* **24**, 1627–42 (2013).

S-11. Terabayashi T, et al. Vascular endothelial growth factor receptor-3 is a novel target to improve net ultrafiltration in methylglyoxal-induced peritoneal injury. *Lab Invest.* **95**, 1029–43 (2015).

S-12. Kato H, et al. Atrial natriuretic peptide ameliorates peritoneal fibrosis in rat peritonitis model. *Nephrol Dial Transplant.* **27**, 526–36 (2012).

S-13. Arai S, et al. Apoptosis inhibitor of macrophage protein enhances intraluminal debris clearance and ameliorates acute kidney injury in mice. *Nat Med.* **22**, 183–93 (2016).
S-14. Maehara N, et al. Circulating AIM prevents hepatocellular carcinoma through complement activation. *Cell Rep.* **9**, 61–74 (2014).
S-15. Strippoli R, et al. Caveolin-1 deficiency induces a MEK-ERK1/2-Snail-1-dependent epithelial-mesenchymal transition and fibrosis during peritoneal dialysis. *EMBO Mol Med.* **7**, 357 (2015).S-14.
S-16. Shi Y, et al. Pristane-induced granulocyte recruitment promotes phenotypic conversion of macrophages and protects against diffuse pulmonary hemorrhage in Mac-1 deficiency. *J Immunol.* **193**, 5129–39 (2014).
S-17. Mizutani M, et al. Connective tissue growth factor (CTGF/CCN2) is increased in peritoneal dialysis patients with high peritoneal solute transport rate. *Am J Physiol Renal Physiol.* **298**, F721–33 (2010).

SUPPLEMENTARY VIDEOS

1: M1-like macrophages engulfed dead cell debris.

2: M2a-like macrophages engulfed dead cell debris.

3: Mesothelial cells engulfed dead cell debris.

Supplementary Videos Files are separately attached.
